# Acute and chronic effects of multivitamin/mineral supplementation on objective and subjective energy measures

**DOI:** 10.1186/s12986-020-00435-1

**Published:** 2020-02-24

**Authors:** F. L. Dodd, D. O. Kennedy, E. J. Stevenson, R. C. Veasey, K. Walker, S. Reed, P. A. Jackson, C. F. Haskell-Ramsay

**Affiliations:** 1 Brain, Performance and Nutrition Research Centre, Northumbria University, Upon-Tyne, Newcastle, NE1 8ST UK; 2 Population Health Sciences Institute, Newcastle University, Newcastle upon Tyne, NE2 4HH UK; 3 Faculty of Health and Life Sciences, Northumbria University, Upon-Tyne, Newcastle, NE1 8ST UK; 4 Department of Psychology, Northumbria University, Upon-Tyne, Newcastle, NE1 8ST UK

**Keywords:** Vitamin, Mineral, Coenzyme Q10, Cognitive, Metabolism, Indirect calorimetry, Energy expenditure, Exercise, Fatigue, Stress

## Abstract

**Background:**

Vitamins and minerals play an essential role within many cellular processes including energy production and metabolism. Previously, supplementation with a multivitamin/mineral (MVM) for ≥28 days resulted in improvements to cognition and subjective state. We have also demonstrated shifts in metabolism during cognitively demanding tasks following MVM in females, both acutely and following 8-week supplementation. The current study aimed to assess these effects further in males and females using metabolically challenging exercise and cognitive tasks.

**Methods:**

The current randomised, placebo-controlled, parallel groups study investigated the effects of a MVM complex in 82 healthy young (18-35y) exercisers. Subjective ratings and substrate metabolism were assessed during 30 min each of increasingly effortful incremental exercise and demanding cognitive tasks. Assessments took place on acute study days following a single dose (Day 1) of MVM, containing 3 times recommended daily allowance of water-soluble vitamins plus CoQ10, and following 4-week supplementation (Day 28).

**Results:**

Energy expenditure (EE) was increased during cognitive tasks following MVM across Day 1 and Day 28, with greater effects in males. In males, MVM also increased carbohydrate oxidation and energy expenditure during exercise across Day 1 and Day 28. In females, mental tiredness was lower during exercise; increases in physical tiredness following 30 min of exercise were attenuated; and stress ratings following cognitive tasks were reduced following MVM. In males, MVM only lowered mental tiredness following 10 min of exercise. These effects were apparent irrespective of day, but effects on mental tiredness were greater on Day 28. Ferritin levels were also higher on Day 28 in those receiving MVM.

**Conclusion:**

These findings extend on existing knowledge, demonstrating increased carbohydrate oxidation and increased energy expenditure in males following MVM supplementation for the first time. Importantly, they show modulation of energy expenditure and subjective tiredness following a single dose, providing further evidence for acute effects of MVM. Differential effects in men and women suggest that sex may play an important role in the effects of MVM on energy metabolism and should be considered in future research.

**Trial registration:**

ClinicalTrials.gov, NCT03003442. Registered 22nd November 2016 – retrospectively registered

## Background

Vitamins and minerals play an essential role within many cellular processes including energy production and anabolic metabolism, which are necessary for normal physical and mental functioning [1, 2]. Deficiencies in both the B vitamins and CoQ10 are associated with fatigue and a number of conditions related to mitochondrial dysfunction [3–6]. Circulating levels of B vitamins have also been shown to be inversely correlated with the potentially neurotoxic amino acid homocysteine, which in turn is positively correlated with cardiovascular disease, age-associated cognitive decline and dementia, although the causality of this relationship is unclear [6, 7].

Exercising in a fasted state is common amongst active individuals, who may do so to avoid discomfort during exercise, or due to time constraints. Fat oxidation is also increased when individuals are fasted prior to exercise as compared to following food [8, 9], providing further impetus for this practice. A negative characteristic of this is that post-exercise mental fatigue has been shown to be higher after exercising in a fasted as compared to a non-fasted state [10]. It has also been proposed that omitting food in this way may increase the risk of experiencing the marginal deficiency of one or more vitamins or minerals observed in the general population of developed countries [11–13]. This is further exacerbated by higher excretion of micronutrients through waste products, such as sweat and urine, during and after strenuous exercise [14]. In addition, biochemical changes and heightened metabolic demands that occur during exercise lead to increases in the requirement for certain micronutrients.

We have recently demonstrated that cognitive tasks are capable of engendering increases in whole-body metabolism and shifts in carbohydrate/fat metabolism similar to those seen during exercise, as measured through indirect calorimetry, alongside increased cerebral blood flow. These metabolic parameters were modulated by micronutrient interventions such that a full spectrum multivitamin/mineral (MVM) with approximately one recommended dietary allowance (RDA) of water soluble vitamins (B vitamins and vitamin C) alongside coenzyme Q10 increased cerebral blood flow after a single dose, and a higher single dose of MVM with approximately 3 RDAs of water soluble vitamins increased fat oxidation and total energy expenditure, with the latter effect still evident after 8 weeks’ supplementation [15]. Previous research has also indicated that chronic supplementation with MVM can improve the performance of cognitive tasks, psychological state or ratings of physical and mental tiredness in adult males and females [16–18]. MVM supplementation has also been shown to reduce homocysteine [16]; levels of which have been shown to both increase and decrease as a consequence of exercise [19–21]. Finally, an acute dose of MVM + guaraná consumed 1 h prior to exercise has been shown to reduce rate of perceived exertion during exercise at 60% VO_2_ max, as compared to placebo in fasted males [22].

Evidence from previous studies of the metabolic and subjective effects of MVM supplementation, coupled with the importance of vitamins and minerals in cellular energy production suggest that enhanced ‘recovery’ from metabolically challenging physical and psychological tasks is possible following acute and chronic supplementation with MVM complex. It is also conceivable that the level of ‘enhanced recovery’ is dependent upon the intensity of the physical and psychological demand encountered. The present study therefore investigated the impact of a single dose and 4 weeks administration of an MVM containing 3 RDAs of water-soluble vitamins plus coenzyme Q10 on subjective ratings of ‘fatigue/stress’, substrate metabolism (as measured with indirect calorimetry) and blood biomarkers of damage, inflammation, cell oxidation and antioxidant activity as a consequence of increasingly effortful incremental exercise, and during metabolically demanding cognitive tasks. Since exercising in a fasted state is common amongst active individuals, a fasted population was employed here. As our previous study only included females, the current study included both males and females and assess any differences in response as a function of sex.

## Methods

### Design

A randomised, placebo-controlled, double-blind, parallel groups design was utilised. Participants attended the lab and were assessed following a single dose (day 1) and after 4 weeks (day 28) supplementation with either Supradyn (3RDA + Co-Q10) (MVM) or matched placebo (PLA). The study was approved by Northumbria University’s Department of Psychology Ethics Committee and was conducted according to the Declaration of Helsinki (1964). The study was registered on clinicaltrials.gov (identifier: NCT03003442).

### Participants

A power calculation was conducted based upon previous indirect calorimetry data showing significant increases to energy expenditure following 8 weeks’ consumption of 3RDA Supradyn [15]. An effect size of f = 0.31 indicated that a total of 86 subjects (43 per arm) with a complete dataset would allow detection of significant effects with a power of 0.8 at 2-sided significant level of 0.05. To allow for dropout, ninety-one male and female volunteers were recruited through opportunity sampling within Newcastle upon-Tyne and the surrounding areas and randomised to treatment. Prior to participation, all participants were required to read an information sheet and provide informed consent. Participants were healthy, non-smokers with a body mass index (BMI) < 30 kg/m^2^ who consumed < 500 mg caffeine per day (from all dietary sources). Participants confirmed that they were not habitually taking any medication (excluding the contraceptive pill) and had not supplemented (within the last month) with vitamins or minerals. All participants self-reported that they were physically active (exercised at least two times per week) and were able to run non-stop on a treadmill at a moderate pace for 30 min. Participants were reimbursed £125 for their time. In a subset of 39 participants, blood samples were taken to assess recovery biomarkers and micronutrient status. These participants were reimbursed £155 for their time.

**Fig. 1:**
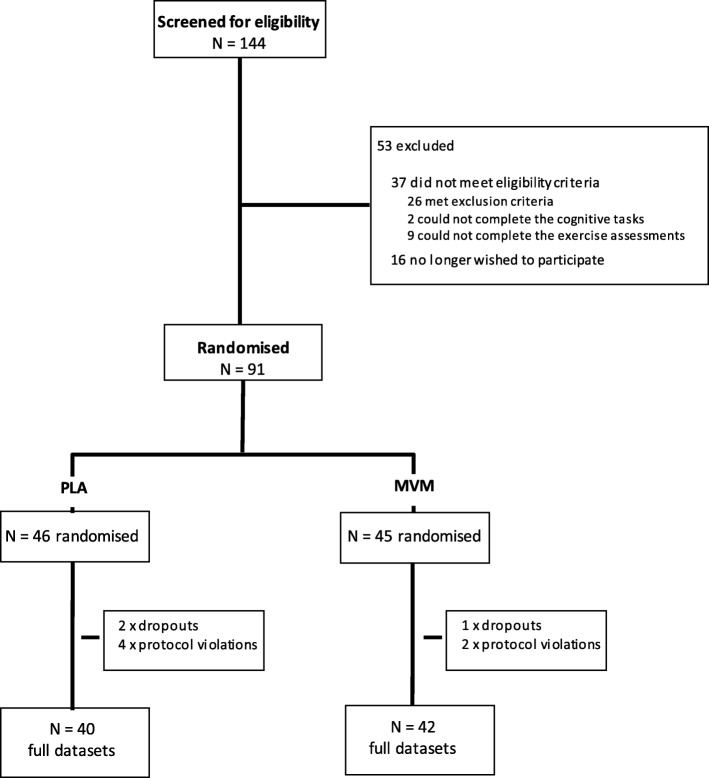
Participant disposition flowchart. PLA = placebo; MVM = multivitamin/mineral

Eighty-two healthy participants aged 18–35 years were included in the per protocol statistical analysis, of these 35 provided blood samples (see Fig. 1).

Participant characteristics for each intervention group can be found in Table 1. There were no significant differences between groups on any of these measures. A subset of 35 participants (PLA = 17 (12 male); MVM = 18 (13 male)) provided blood samples included in analyses of biomarkers.

**Table 1:** Baseline characteristics for each intervention group (mean ± standard deviation)

Measure	Treatment	Mean	SD
Sex male/female	PLA	25/15	
	MVM	25/17	
Age at enrolment	PLA	22.3	3.35
	MVM	23.7	4.61
Years in education	PLA	16.6	2.53
	MVM	16.3	2.97
Alcohol consumption (units/day)	PLA	0.75	0.86
	MVM	1.07	1.28
Caffeine consumption (mg/day)	PLA	107	93
	MVM	117	105
Number of fruit and veg portions per day	PLA	3.60	1.59
	MVM	3.42	1.52
IPAQ score	PLA	4424	3522
	MVM	4755	3238
BMI (kg/m^2^)	PLA	23.1	2.62
	MVM	23.9	2.76
Energy expenditure (Kcal/day)	PLA	1491.5	342.4
	MVM	1601.7	374.2

*PLA* placebo, *MVM* multivitamin/mineral, *mg* milligrams, *IPAQ* international physical activity questionnaire, *BMI* body mass index, *Kcal* kilocalories, *kg* kilograms, *m*^*2*^ metres squared

### Treatment

Participants were randomly allocated to receive either 28 days of Supradyn (3RDA + Co-Q10) (MVM) or matched placebo (PLA). Details of treatment composition can be found in Table 2. Treatments were randomised by the manufacturer separately for males and females in blocks of four. Treatments were prepared and bottled by the manufacturer in accordance with a computer-generated randomisation list and delivered to the investigational site identified only by their randomisation code (sealed emergency code break envelopes were provided to site with treatments). Males and females were allocated sequentially to their respective randomisation code lists. Treatments were provided in single bottles and were identical in appearance to ensure participants remained blind to the treatment they had received. Participants were directed to take one tablet with water, daily for a period of 28 days. Compliance was assessed via treatment diaries and pill counting.

**Table 2:** Composition of multivitamin/mineral - Supradyn (3RDA + CoQ10) active Ingredients

Active	Units	Amount per tablet	% of RDA (EU)
Vitamin A	μg	800	100
Vitamin B1	mg	3.3	300
Vitamin B2	mg	4.2	300
Vitamin B6	mg	2	143
Vitamin B12	μg	3	120
Vitamin C	mg	180	225
Vitamin D3	μg	5	100
Vitamin E	mg	12	100
Vitamin K1	μg	25	33
Biotin	μg	50	100
Folic acid	μg	200	100
Niacin (Nicotinamide)	mg	48	300
Pantothenic acid	mg	18	300
Calcium	mg	120	15
Copper	mg	1	100
Iodine	μg	150	100
Iron	mg	14	100
Magnesium	mg	80	21
Manganese	mg	2	100
Molybdenum	μg	50	100
Selenium	μg	50	91
Zinc	mg	10	100
Coenzyme Q10	mg	4.5	–

### Measures

#### Energy metabolism - indirect calorimetry

During the exercise element of the study and during completion of cognitive tasks, pulmonary (minute) ventilation, oxygen uptake (VO_2_), carbon dioxide production and respiratory exchange ratio were determined from breath-by-breath expired gases, measured using indirect open circuit calorimetry (Cortex Metalyzer 3B, Cranlea, UK). Participants breathed as normal into a mask that covered the nose and mouth, which was connected using falconia tubing to the Metalyzer. This data was then used to determine measures of energy expenditure (kcal/min), carbohydrate oxidation (g/min) and fat oxidation (g/min), using standard formulae, as described by Frayn [23].

#### Subjective tiredness and exertion

##### Mental and physical tiredness

At baseline, during exercise and during cognitive tasks, visual analogues scales were presented using the Computerised Mental Performance Assessment System (COMPASS, Northumbria University, Newcastle upon Tyne, UK). Line scales anchored “not at all” (left-end) and “extremely” (right-end) were presented to participants and they were required to mark a cross on a line using a mouse (baseline and during cognitive tasks) or touch-screen tablet (during exercise) to indicate their current level of mental and physical tiredness. These were scored as % along the line from left to right.

Ratings of perceived exertion (RPE).

During exercise, RPE [24] measurements were obtained, using the BORG category 20 scale. Participants verbally rated their exertion as being between 6 (no exertion at all) and 20 (maximal exertion).

#### Subjective stamina, concentration and stress

At baseline and during cognitive tasks, visual analogues scales (VAS) were presented on a laptop computer using COMPASS. Participants were asked to use a mouse to mark a cross on a line to indicate their current levels of “mental stamina”, “physical stamina” and “concentration” on scales anchored ‘“very low” (left-end) and “very high” (right-end); and “stress” on a scale anchored “not at all” (left end) and “extremely” (right end). All visual analogue scales were scored as % along the line from left to right.

### Blood samples

#### Recovery biomarkers

##### Blood collection and storage

Venous blood samples were collected at both the acute and chronic assessments from an inlaying cannula (22-gauge, B Braun, Germany) in the median cubital vein of the non-dominant arm. Blood samples (15 ml) were collected at baseline, at rest post-exercise, following cognitive tasks (1 h 45 min post-exercise), and at 24 and 48 h post-exercise to assess recovery from exercise. Samples were collected in BD vacutainers© (Medisave, UK) pre-prepared with Ethylenediaminetetraacetic acid (EDTA) or pre-prepared serum-separating tubes (SST). Those collected in ETDA vacutainers (10 ml) were inverted several times after collection to prevent coagulation and refrigerated immediately at 4 °C for up to 6 h. Whole blood samples treated with EDTA were centrifuged (4 °C, 3000 rpm, 10 min) (Beckman Coulter, Allegra X-22R, USA). Plasma was then extracted and 1.0 ml aliquoted into 3 × 1.5 ml separate Eppendorf© (Starstedt, Germany) tubes for analysis of protein carbonyl (Cayman Chemical, USA), interleukin 6 (IL-6) (Abcam, UK) and glutathione peroxidase (GPX) (Cayman Chemical, USA). For F-2 isoprostanes (Cayman Chemical, USA) analysis, 1.0 ml plasma was aliquoted into 1 x pre-prepared butylated hydroxytoluene (BHT) (stored in − 20 °C freezer) Eppendorf© tube. Blood samples collected in SST vacutainers (5 ml) were refrigerated immediately at 4 °C and at least 30 min was allowed for clotting. Whole blood samples were centrifuged (room temperature, 2800 rpm, 5 min). Serum was extracted immediately in a dark lab and 0.5 ml aliquoted into 1 x false bottomed tube (FBT) (Roche Diagnostics, Germany) for C-reactive protein (CRP) (CRPL3, Roche Cobas c702, Germany) analysis by Newcastle Laboratories (Freeman Hospital, UK). All samples were stored at − 80 °C until analysis. Unless specified, biomarker analysis was conducted within the Faculty of Health and Life Sciences at Northumbria University.

#### Micronutrient status and homocysteine levels

##### Blood collection and storage

Venous blood samples (7.7 ml) were collected at baseline (acute study day only) and at rest post-exercise at both the acute and chronic assessments (as per recovery biomarkers), to assess micronutrient status. Samples were collected in BD vacutainers©, pre-prepared with sodium citrate or SST. Blood samples collected in SST vacutainers (5 ml) were processed as per recovery biomarkers, above with serum extracted in a dark lab and 2.5 ml aliquoted into 1 x FBT for analysis of serum ferritin (Roche Cobas e602, Germany), vitamin B12 (B12) (Roche Elecsys, Germany) and creatinine (CREP2, Roche Cobas c702, Germany). Blood samples collected in sodium citrate vacutainers (2.7 ml) were inverted 5–6 times then refrigerated immediately at 4 °C for at least 30 min. Whole blood samples were centrifuged (room temperature, 2278 rpm, 10 min). Plasma was extracted and 1.35 ml aliquoted into 1 x FBT for homocysteine (Werfen TOP 700, Aus) analysis. All samples were stored at − 80 °C until analysis. Analysis was conducted by Newcastle Laboratories (Freeman Hospital, UK).

##### Cognitive measures

All cognitive tasks were conducted via laptop computer using the Computerised Mental Performance Assessment System (COMPASS, Northumbria University, Newcastle upon Tyne, UK). COMPASS is a software platform for the presentation of classic and bespoke computerised cognitive tasks which has previously been successful in identifying cognitive effects of nutritional interventions [25–27]. The ‘Cognitive Demand Battery’ (CDB) (2 min each of serial 3s subtractions and serial 7s subtractions and a 5 min rapid visual information processing (RVIP) task, repeated 6 times) was utilised, that has previously been shown to be sensitive to a number of nutritional interventions [28, 29], including multivitamins [18]. In the present study, a shorter 30-min version of this battery was employed, where participants were required to repeat each of the tasks 3 times. On-screen instructions were provided prior to each task and the participant controlled their own progress throughout.

Serial subtractions (3s and 7s) (2 min).

A randomly generated starting number between 800 and 999 appeared on the screen and participants were instructed to count backwards as quickly and as accurately as possible from this number in threes (or sevens), using the keyboard’s linear number keys to make their response. Each digit was represented on screen by an asterisk and responses were cleared when the ‘enter’ key was pressed. Participants were only shown one number on screen and the rest of the numbers were generated by subtracting from the previous number in their head. In the case of incorrect responses, subsequent responses were scored as positive if they were correct in relation to the new number. The tasks were scored for total responses and errors.

Rapid Visual Information Processing (RVIP) (5 min).

A continuous series of digits between 1 and 9 were presented on-screen one at a time at the rate of 100 per minute in a pseudo-random order and participants were required to identify 3 consecutive odd or 3 consecutive even digits. The participant responded to the detection of a target string by pressing the centre button on a response pad as quickly as possible. The task was continuous with 8 correct target strings being presented in each minute. This task was scored for percentage of target strings correctly detected, average reaction time for correct detections, and number of false alarms.

##### Procedure

Participants were required to attend the Brain, Performance and Nutrition Research Centre (Northumbria University) for 4 separate sessions:

Visit 1 - Screening/training.

The first visit was a screening and training session, which comprised obtaining informed consent and confirmation of eligibility to participate (review of inclusion and exclusion criteria). This included a review of medical history and vital signs including blood pressure, heart rate and height and weight measures (for determination of BMI) and completion of a caffeine consumption questionnaire. Demographic data was also collected and participants were required to document their exercise routine using the shortened version of the International Physical Activity Questionnaire (IPAQ) [30]. Eligible participants were then trained on the cognitive tasks they would be completing as part of the study. In the second half of the session, participants completed two preliminary tests in order to determine: 1) the relationship between maximal oxygen consumption (VO_2_ max) and running speed on a flat treadmill using a 16-min test; 2) their VO_2_ max using an incremental treadmill test whereby the gradient was increased by 1%·min^− 1^ to exhaustion, as previously described in full detail [31].

Visit 2 - Exercise familiarisation.

At least 48 h following Visit 1, participants attended the lab to complete a familiarisation run and to provide confirmation, and opportunity for adjustment, of the running speeds required to achieve 60, 65, 70, 75, 80 and 85% VO_2_ max. If average VO_2_ was > 2.5% above or below that calculated to be the required VO_2_, speed adjustments were then made at the discretion of the researcher.

Visit 3 - Day 1 - Acute assessment.

**Fig. 2:**
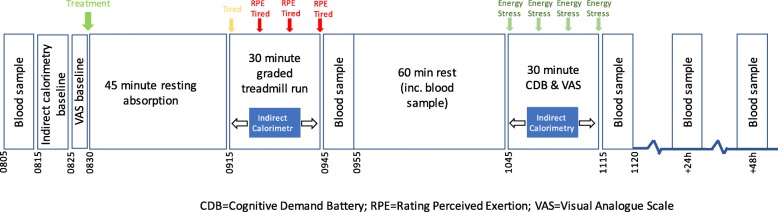
Schematic showing acute study trial day procedures

Within 14 days of visit 2, participants attended the lab in the morning having refrained for 24 h from strenuous exercise, alcohol, and the intake of analgesic and other OTC medication. Systemic anti-histamines were avoided for 48 h. Participants were required to fast for 10 h prior to their visit and to record their food intake (via food diary) over the preceding 24 h. Continued conformity with the inclusion and exclusion criteria was reviewed, caffeine consumption, exercise routine, concomitant medication and general health were documented. A weight review was also conducted. The subset of participants provided a venous blood sample (protein carbonyls, F2-isoprostanes, GPX, IL-6, CRP, B12, ferritin, homocysteine, creatinine) then all participants provided a baseline indirect calorimetry measure (10 min) and baseline subjective visual analogue ratings (mental tiredness, physical tiredness, mental stamina, physical stamina, concentration, stress). Participants were then randomised to treatment, before resting in the laboratory for 45 min. At 45 min post-dose, participants completed a 30-min treadmill run of graded intensity; 20 min at 60% VO_2_ max and 2 min each at 65, 70, 75, 80 and 85% VO_2_ max. Indirect calorimetry, physical tiredness, mental tiredness and perceived exertion data were collected prior to (with the exception of RPE), at 10 min during, and after the treadmill run. Participants providing blood samples then did so (protein carbonyls, F2-isoprostanes, GPX, IL-6, CRP, B12, ferritin, homocysteine, creatinine) and all participants rested in the laboratory for approximately 1 h. At approximately 2 h 15 min post dose, the cognitive and mood assessment was completed. Indirect calorimetry was continually recorded during this assessment. Following the cognitive tasks, a final blood sample (protein carbonyls, F2-isoprostanes, GPX, IL-6, CRP) was taken from the relevant subset. During rest periods, participants were required to remain in the laboratory, but were allowed to read, write or watch television. Water intake was recorded during this visit, so it could be replicated during visit 4. At 24, and 48, hours post-exercise and having fasted for 10 h prior, those participants who had elected to provide blood samples returned. See Fig. 2 for schematic depicting Visit 3, including blood samples for the subset that elected to participate.

Visit 4 (Day 28 - Chronic Assessment).

Visit 4 took place 28 days after visit 3 and was identical with the exception that no treatment was administered, there was no absorption period and no baseline measurements were taken. Therefore, participants arrived at 9 am, gave a blood sample (if applicable) and then commenced the exercise protocol. Participants were asked to consume the same or similar foods in the 24 h period prior to visit 4 as recorded in the food diary prior to visit 3. Food intake was comparable across both visits and no changes to caffeine consumption, exercise routine or general health were reported during the intervention period. After completion of all trial procedures participants were asked to guess which treatment they believed they had received and were then fully debriefed.

##### Statistics

All post dose outcome measures were modelled using the MIXED procedure in SPSS (version 24.0, IBM corp.). Analysis of RPE included the term ‘treatment’ and all possible interactions with ‘sex’, ‘day’ and ‘repetition’ as fixed factors. The model for exercise indirect calorimetry and visual analogue scales was identical to that for RPE with the addition of the respective baseline values. Analysis of cognition included the term ‘treatment’ and all possible interactions with ‘sex’, ‘day’, ‘repetition’ and ‘task’ as fixed factors. The model for indirect calorimetry data collected during cognitive tasks was identical with the addition of the respective baseline values. Analyses of micronutrient status and homocysteine levels included the terms ‘treatment, ‘treatment * day’ interactions and the respective baseline values as fixed factors. Analyses of recovery biomarkers included the terms ‘treatment’, and all possible interactions with ‘day’, and ‘sample’, as well as the respective baseline values as fixed factors. Significant effects (*p* < 0.05) were followed up with Bonferroni-corrected pairwise comparisons. Only those effects showing a significant difference between treatments on the initial analysis are reported. Chi-squared was used to assess treatment guess responses.

## Results

### Indirect calorimetry

#### Carbohydrate oxidation during exercise

**Fig. 3:**
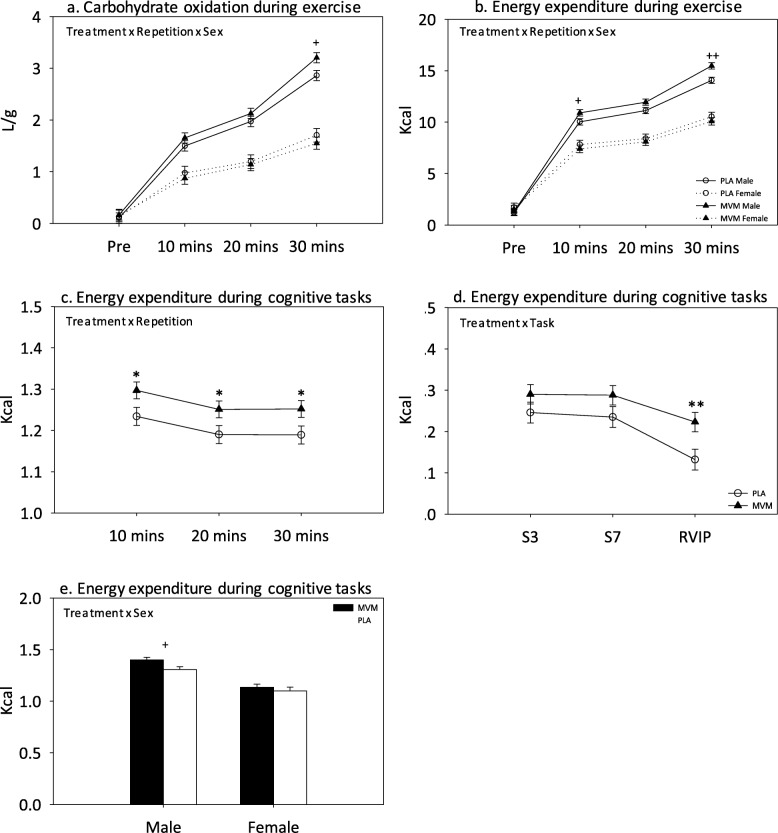
Effects of intervention on (**a**) carbohydrate oxidation during exercise, (**b**) energy expenditure during exercise and, (**c-e**), energy expenditure during cognitive tasks. PLA = placebo; MVM = multivitamin/mineral; S3 = serial 3 subtractions; S7 = serial 7 subtractions; RVIP = rapid visual information processing; * = *p* < 0.05; ** = *p* < 0.01; + = *p* < 0.05 in males; ++ = *p* < 0.01 in males

Significant treatment x repetition [F (6, 471.3) = 276.9, *p* < 0.0001]; treatment x sex [F (2, 102.2) = 29.8, *p* < 0.0001]; and treatment x repetition x sex [F (6, 471.3) = 29.9, *p* < 0.0001] (see Fig. 3a) interactions were observed. Pairwise comparisons revealed significantly higher carbohydrate oxidation following 30 min of exercise in males receiving MVM compared to PLA (*p* = 0.015) across both the acute (Day 1) and chronic (Day 28) visits.

#### Energy expenditure during exercise

Significant treatment x repetition [F (6, 485.7) = 1127.9, *p* < 0.0001]; treatment x sex [F (2, 100.3) = 28.6, *p* < 0.0001]; and treatment x repetition x sex [F (6, 485.7) = 55.4, *p* < 0.0001] (see Fig. 3b) interactions were observed. Pairwise comparisons revealed significantly higher energy expenditure in males receiving MVM compared to PLA (10 mins: *p* = 0.04; 20 mins: *p* = 0.057; 30 mins: *p* = 0.001) across both the acute (Day 1) and chronic (Day 28) visits.

#### Energy expenditure during cognitive tasks

A significant main effect of treatment [F (1, 142.9) = 4.82, *p* = 0.03]; treatment x repetition [F (4, 1146.8) = 10.4, *p* < 0.0001] (see Fig. 3c); treatment x task [F (4, 1044.1) = 9.71, *p* < 0.0001] (see Fig. 3d); and treatment x sex [F (2, 144.1) = 25.5, *p* < 0.0001] (see Fig. 3e) interactions were observed. Pairwise comparisons revealed significantly higher energy expenditure following MVM compared to PLA during each repetition of the CDB irrespective of task (10 mins, *p* = 0.036; 20 mins: *p* = 0.044; 30 mins: *p* = 0.035); during the RVIP task irrespective of repetition (*p* = 0.009); and in males irrespective of task or repetition (*p* = 0.011). These effects were apparent across both the acute (Day 1) and chronic (Day 28) visits.

### Subjective state visual analogue scales

#### Mental tiredness during exercise

**Fig. 4:**
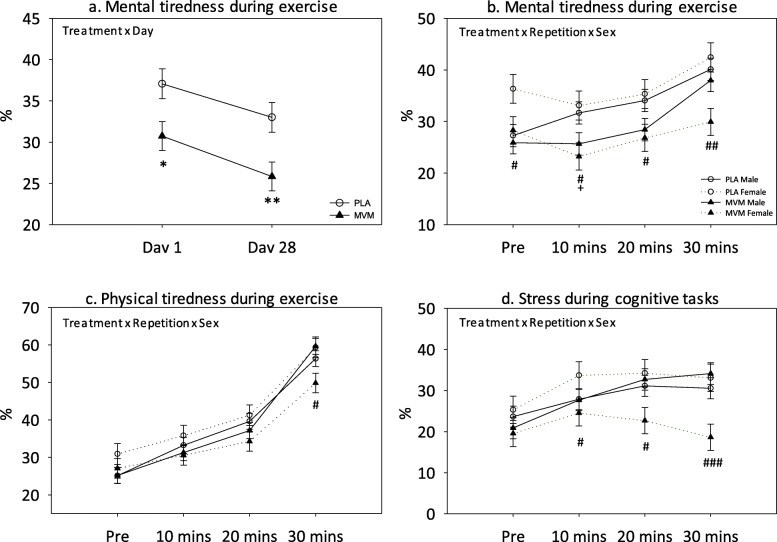
Effects of intervention on (**a-b**) mental tiredness during exercise, (**c**) physical tiredness during exercise and, (**d**) stress during cognitive tasks. PLA = placebo; MVM = multivitamin/mineral; * = *p* < 0.05; ** = *p* < 0.01; # = *p* < 0.05 in females; ## = *p* < 0.01; ### = *p* < 0.005 in females; + = *p* < 0.05 in males

A significant main effect of treatment [F (1, 106.2) = 10.7, *p* = 0.001]; treatment x day [F (2, 364) = 4.93, *p* = 0.008] (see Fig. 4a); treatment x repetition [F (6, 492.8) = 12.8, *p* < 0.0001]; and treatment x repetition x sex [F (6, 492.8) = 3.70, *p* = 0.001] (see Fig. 4b) interactions were observed. Pairwise comparisons of the treatment x day effect revealed significantly lower mental tiredness ratings following MVM compared to PLA on Day 1 (*p* = 0.013) and Day 28 (*p* = 0.005). Pairwise comparisons of the treatment x repetition x sex effect revealed lower ratings following MVM compared to PLA in males following 10 min of exercise (*p* = 0.049), with a trend towards the same at 20 min (*p* = 0.064) and in females prior to (*p* = 0.036) and during exercise (10 mins: *p* = 0.01; 20 mins: *p* = 0.026; 30 mins: *p* = 0.001).

#### Physical tiredness during exercise

A significant treatment x repetition x sex interaction [F (6, 484.9) = 2.37, *p* = 0.029] was observed for ratings of physical tiredness (see Fig. 4c). Pairwise comparisons revealed significantly lower ratings following 30 min of exercise in females receiving MVM compared to PLA (*p* = 0.012).

#### RPE during exercise

A significant treatment x repetition interaction [F (4, 365.6) = 376.8, *p* < 0.0001] was observed on RPE ratings. Pairwise comparisons revealed significantly lower ratings following 10 min of exercise in MVM compared to PLA (*p* = 0.030), with a trend towards the same following 20 min (*p* = 0.063).

#### Stress during cognitive tasks

Significant treatment x repetition [F (6, 502.3) = 7.26, *p* < 0.0001]; and treatment x repetition x sex [F (6, 502.3) = 2.86, *p* = 0.009] interactions were observed (see Fig. 4d). Pairwise comparisons revealed significantly lower stress ratings following each repetition of the CDB (10 mins, *p* = 0.046; 20 mins: *p* = 0.012; 30 mins: *p* = 0.002) in females receiving MVM compared to PLA.

### Recovery biomarkers

*IL-6:* Due to the majority of samples showing values below the level of detection, the analysis of IL-6 only included 6 participants who all had multiple samples below the detection level. This result is, therefore, not reported.

### Micronutrient status and homocysteine levels

*Ferritin:* A significant treatment x day effect was observed [F [2, 32]=4.73, *p* = 0.016], which pairwise comparisons revealed was due to significantly higher ferritin levels following MVM than PLA on Day 28 (*p* = 0.03).

Unadjusted means and standard deviations for all outcomes can be found in Supplementary Tables S1–S8 [see Additional file 1].

### Treatment guess

Success of blinding was confirmed via treatment guess at the end of the study. Sixty-two percent of participants in the placebo group and 73% of participants in the MVM group believed they had received placebo. Chi-squared analysis confirmed this to be a non-significant difference (*p* = 0.267).

## Discussion

The current study explored the acute (Day 1) and repeated dose (Day 28) effects of a multivitamin/mineral (MVM) supplement on energy metabolism and subjective state during incremental exercise and metabolically demanding cognitive tasks. In males, MVM led to increased carbohydrate oxidation in the final 10 min of an incremental treadmill run as well as increased energy expenditure during each 10-min block of exercise across both Day 1 and Day 28. Energy expenditure was also increased during each repetition of the cognitively demanding tasks following MVM for both males and females across Day 1 and 28, with specific effects also observed during the RVIP task irrespective of repetition. MVM also led to increased energy expenditure in males irrespective of task, repetition or day. Those receiving MVM reported lower subjective RPE following 10 min of exercise, with a trend towards the same following 20 min across both Day 1 and Day 28. In females, mental tiredness ratings were lower at each of the 4 time points measured during exercise; increases in physical tiredness following 30 min of exercise were attenuated; and stress ratings following each repetition of the cognitively demanding tasks were reduced following MVM as compared to placebo. In males, MVM led to lower mental tiredness following 10 min of exercise with a trend towards the same following 20 min. These effects were irrespective of whether measured on Day 1 or Day 28, with the exception of an interaction effect on mental tiredness showing that although mental tiredness was reduced following MVM compared to placebo on Day 1, this reduction was greater on Day 28. Ferritin levels were also higher on Day 28 in those receiving MVM.

These findings indicate that MVM supplementation can increase carbohydrate oxidation and energy expenditure during incremental treadmill running and increase energy expenditure during the performance of cognitively demanding tasks. This supports previous demonstrations of increased energy expenditure following 26 weeks of MVM supplementation in obese females at rest [32] and following both a single dose and repeated doses (56 days) of MVM in females during cognitively demanding tasks [15]. Our results indicate that increases in energy expenditure following MVM supplementation are observed during exercise and not only extend to, but are greater in, males. The increase in carbohydrate oxidation following MVM is a novel finding, which could indicate changes in the intracellular and extracellular metabolic environments, increased carbohydrate availability or increased exercise intensity [33]. As this effect was only observed during the final 10 min of exercise when the intensity increased incrementally from 65 to 85% VO_2_ max, and as the reliance on fat oxidation typically decreases when exercising in excess of 65% VO_2_ max [34], this indicates a specific upregulation of carbohydrate oxidation when reliance on it would be highest. As carbohydrate availability is often the rate-limiting factor in high intensity and endurance exercise [35], this finding of increased carbohydrate oxidation together with increased energy expenditure throughout the run, may indicate the potential for performance-enhancement following MVM. A previously observed increase in fat oxidation during cognitive tasks was not demonstrated here, which possibly relates to the inclusion of an exercise paradigm prior to cognition that may have depleted fatty acid availability.

Whilst MVM-related increases in energy expenditure and carbohydrate oxidation were not observed in females during exercise, subjective tiredness ratings were modulated. Female ratings of mental tiredness were lower at each time point during, and physical tiredness was lower immediately following, exercise when compared to placebo. Males supplemented with MVM only showed significant reduction in mental tiredness following 10 min of exercise, with RPE also lower at this time point for both sexes. These findings with regards female subjective ratings mirror those for male energy metabolism, potentially suggesting that whilst males expended more energy, females may have conserved their energy but reported lower tiredness instead. Females receiving MVM also reported lower subjective stress when rated following each repetition of the CDB. Mood improvements following MVM supplementation, such as reduced stress and fatigue, are well-documented in both men and women [7, 16, 36, 37], including in men following the original 60-min version of the CDB [18].

Greater effects on indirect calorimetry were observed in males. The simplest explanation of this sex difference is that, as expected, the males in the sample exhibited significantly higher overall metabolism during both exercise and cognitive task performance, and this factor may underpin the results. However, females are known to have a higher percentage of body fat than males [38], a factor which has been shown to influence substrate oxidation. Venables et al. [39] demonstrated that during exercise of increasing intensity (in the absence of MVM supplementation) a sex dimorphism exists, whereby lipids contribute to a higher proportion of energy expenditure in women than in males. Given this greater contribution of lipids to oxidative metabolism in women it is possible that delayed dependency on carbohydrate oxidation during incremental exercise led to the lack of effects on MVM on this outcome in women. The absence of an effect on fat oxidation in the present study despite the inclusion of a female cohort may be due to sample size. In addition, since energy expenditure and substrate metabolism have been shown to be influenced by menstrual cycle phase due to fluctuating levels of oestrogen and progesterone and their effects on metabolic pathways [40, 41], it is possible that effects were not detected in women due to a lack of control for this variable. However, effects on substrate metabolism were observed previously as a consequence of MVM supplementation despite not controlling for this [15] and examination of standard deviations does not indicate greater variation in response in females. One of the reasons for employing a physically active sample using an exercise paradigm was that exercise has been shown to decrease nutrient status [14]. It is possible that males were suffering a greater deficiency, than women, in one or more of the micronutrients relevant to energy metabolism, and this has driven the sex dimorphism on these measures. Whilst this proposition is contrary to sex differences in deficiencies at a national level, it is important that nutritional status is confirmed in future studies.

In terms of sex differences in subjective response, it is perhaps surprising that differential sex effects of MVM on tiredness and stress were observed in the current study since these effects have previously been demonstrated in males. However, mental tiredness and stress ratings were higher in women than men at baseline, which may have increased their susceptibility to mood modulation. This observation is in line with the higher prevalence of depression [42] and anxiety disorders [43] in women generally when compared to males. Higher levels of negative affect in response to an induced stressor in females when compared to males have also been reported [44], which may be relevant here given the increased stress seen following completion of the cognitive demand battery. As both mental tiredness and stress ratings have been shown to be inversely related to iron status [45, 46], the higher baseline ratings in females observed here may relate to the higher prevalence of iron deficiency in females seen nationally [12]. Ferritin levels for the subset who provided blood samples confirm that whilst males were sufficient at baseline, females showed marginal deficiency (data not shown). Therefore, the effects in females may reflect a correction of this insufficiency following intervention. Interestingly, differential gender-specific dietary patterns have also been identified, which suggest that women may be more susceptible to mental distress in response to nutritional insufficiency [47]. Further exploration of differential sex effects of MVM are required given the differences in micronutrient requirements between men and women and the higher deficiencies of a range of micronutrients in women.

Importantly the findings of the current study indicate that metabolic and psychological effects of MVM are apparent across both the first day of treatment and after 28 day’s treatment. This evidence for acute effects of MVM strengthens the findings from earlier research demonstrating modulation of both physiological and subjective parameters. Positive effects of a single dose of MVM on metabolism (fat oxidation and energy expenditure) and cerebral blood flow [15], and functional brain activity [48, 49] have been demonstrated during the performance of cognitive tasks. Combined with guaraná, psychological effects have also been observed during exercise, where an acute dose of MVM led to attenuation of subjective ratings of exhaustion [22]. Of particular interest here, the findings of the aforementioned study were, in part, attributed to the low caffeine content of the guaraná component and/or a synergy between the two. Findings from the present study of an attenuation of perceived exhaustion after the first 10 min of exercise with a trend towards the same at 20 min would suggest that this effect was largely driven by the MVM. Whilst this study provides further evidence for effects of MVM following a single dose it also reinforces the positive benefit of chronic supplementation with MVM. MVM-related reductions in mental tiredness were observed on both days; however, these effects were greater following 28 days supplementation. This may reflect the cumulative effect of MVM supplementation and time taken for body stores to be replenished [4].

Previously we have shown acute effects on energy expenditure with an MVM that did not contain CoQ10 [15] indicating that the effect is not dependent on this coenzyme. As our previous study failed to find effects of a lower dose (1 RDA of water-soluble vitamins) MVM with CoQ10 on carbohydrate oxidation, it is unclear if this effect relates to the inclusion of CoQ10 or to the higher dose (3 RDAs) of water-soluble vitamins. It is also possible that this effect is exercise-specific and only observed at higher intensity exercise, since in the aforementioned study measurements were taken during performance of cognitive tasks [15]. With regards the vitamin and mineral content of the MVM, these compounds are involved in a number of catabolic and anabolic processes important for cellular functioning at the physiological and neurological level [4]. Specifically in relation to energy metabolism, B vitamins in particular play a key role in mitochondrial energy production and are necessary co-factors and co-enzymes within the citric acid cycle in its synthesis of adenosine triphosphate (ATP) [1, 3, 6]. Similarly, minerals including calcium, iron and magnesium are fundamental in the metabolism of ATP; as mitochondrial carriers, in the facilitation of electron transfer and as components of enzymatic reactions [4, 50, 51]. Although it is not possible to identify specific compounds responsible for the effects observed here, it is probable that supplementation of a full spectrum of vitamins and minerals in the form of a MVM is necessary to ensure differing deficiencies across participants are eliminated. This is particularly relevant when it is considered that even in developed countries a noteworthy proportion of the population consume a diet with suboptimal levels of micronutrients [12, 52, 53].

The absence of effects on indices of recovery suggests that the MVM was not effective in modulating these parameters. This is perhaps unsurprising given the healthy status of the participants and the nature of the exercise paradigm employed. Turning to the effects on micronutrient status, there was a significant increase in ferritin following MVM as compared to placebo on Day 28. This finding is as expected given that chronic iron supplementation is known to increase ferritin levels [54]. With regards to homocysteine, previous research has demonstrated a reduction in this amino acid as a consequence of MVM supplementation following demanding cognitive tasks [15, 16]. In the current study although there was a trend towards reduced homocysteine following MVM as compared to placebo, this did not reach significance. This may reflect the younger and fitter sample employed here.

Strengths and limitations.

The use of indirect calorimetry to measure metabolism is a strength of this study. The inclusion of both physical and mental demand enabled the detection of differential effects of MVM on metabolism under these differing conditions. The study design also allowed for the acute (as well as chronic) physiological and subjective effects of MVM to be detected. For a study comparing the effects of sex, the sample size of males and females was relatively small. A larger sample may have provided a clearer picture of the differences between men and women in terms of effects of MVM on metabolism. In addition, only a subset of participants were required to provide blood samples, from which only 35 full data sets were obtained. Furthermore with regards blood sampling, baseline nutritional status was not established at the outset of the study, therefore no conclusions could be drawn on the significance of this factor on the outcomes measured. A further limitation which may have impacted the ability to detect cognitive effects was the absence of baseline (pre-treatment) cognitive performance.

## Conclusions

The results of the current study provide further support for the acute effects of MVM on energy metabolism and subjective tiredness but also highlight the cumulative effect of chronic supplementation. Increased energy expenditure and a novel finding of increased carbohydrate oxidation during exercise were observed in men, with a reduction in subjective tiredness observed predominantly in women. These findings were demonstrated in young adults with normal BMI and moderate activity level and should only be extrapolated to other populations with caution. It is also important to note that the lack of effects on substrate metabolism during exercise in women indicate that a large proportion of the sample did not show efficacy on these measures. These findings suggest that sex may play an important role in the effects of MVM on energy metabolism and should be considered in future research. Whilst these effects on energy metabolism and subjective energy are important, further work is needed to determine their clinical relevance. Future research should also explore whether the effects on energy demonstrated here can be achieved through dietary modulation rather than supplementation.

## Supplementary information

**Additional file 1: Table S1.** Indirect calorimetry during exercise. **Table S2.** Indirect calorimetry during cognitive tasks. **Table S3.** Tiredness ratings during exercise and cognitive tasks. **Table S4.** Rating of Perceived Exertion during exercise. **Table S5.** Energy and stress ratings during cognitive tasks. **Table S6.** Cognitive task performance. **Table S7.** Micronutrient, creatinine and homocysteine levels. **Table S8.** Recovery biomarker levels.

## Data Availability

The datasets analysed during the current study are available from the corresponding author on reasonable request.

## Abbreviations

ATP: Adenosine triphosphate
B12: Vitamin B12
BHT: Butylated hydroxytoluene
BMI: Body mass index
CDB: Cognitive demand battery
COMPASS: Computerised mental performance assessment system
CoQ10: Coenzyme Q10
CRP: C-reactive protein
EDTA: Ethylenediaminetetraacetic acid
FBT: False bottom tube
GPX: Glutathione peroxidase
IPAQ: International physical activity questionnaire
MVM: Multivitamin/mineral
PLA: Placebo
RDA: Recommended dietary allowance
RPE: Ratings of perceived exertion
RVIP: Rapid visual information processing
SST: Serum separating tube
VAS: Visual analogue scales
VO_2_ max: Maximal oxygen consumption
VO_2_: Oxygen uptake

## References

[1] Depeint F, Bruce WR, Shangari N, Mehta R, O'Brien PJ (2006). Mitochondrial function and toxicity: role of the B vitamin family on mitochondrial energy metabolism. Chem Biol Interact.

[2] Yuen AW, Sander JW (2011). Impaired mitochondrial energy production: the basis of pharmacoresistance in epilepsy. Med Hypotheses.

[3] Depeint F, Bruce WR, Shangari N, Mehta R, O'Brien PJ (2006). Mitochondrial function and toxicity: role of B vitamins on the one-carbon transfer pathways. Chem Biol Interact.

[4] Huskisson E, Maggini S, Ruf M (2007). The role of vitamins and minerals in energy metabolism and well-being. J Int Med Res.

[5] Dai Y-L, Luk T-H, Yiu K-H, Wang M, Yip PM, Lee SW (2011). Reversal of mitochondrial dysfunction by coenzyme Q10 supplement improves endothelial function in patients with ischaemic left ventricular systolic dysfunction: a randomized controlled trial. Atherosclerosis.

[6] Kennedy D (2016). B vitamins and the brain: mechanisms, dose and efficacy—a review. Nutrients..

[7] Kennedy DO, Haskell CF (2011). Vitamins and cognition. Drugs.

[8] Backhouse SH, Williams C, Stevenson E, Nute M (2007). Effects of the glycemic index of breakfast on metabolic responses to brisk walking in females. Eur J Clin Nutr.

[9] Gonzalez JT, Veasey RC, Rumbold PL, Stevenson EJ (2013). Breakfast and exercise contingently affect postprandial metabolism and energy balance in physically active males. Br J Nutr.

[10] Veasey RC, Gonzalez JT, Kennedy DO, Haskell CF, Stevenson EJ (2013). Breakfast consumption and exercise interact to affect cognitive performance and mood later in the day. A randomized controlled trial. Appetite.

[11] Adams JS, Hewison M (2010). Update in vitamin D. J Clin Endocrinol Metab.

[12] Ruston D (2004). The national diet and nutrition survey: adults aged 19 to 64 years: nutritional status (anthropometry and blood analytes), blood pressure and physical activity: stationery office.

[13] Schleicher RL, Carroll MD, Ford ES, Lacher DA (2009). Serum vitamin C and the prevalence of vitamin C deficiency in the United States: 2003–2004 National Health and nutrition examination survey (NHANES). Am J Clin Nutr.

[14] Manore MM (2000). Effect of physical activity on thiamine, riboflavin, and vitamin B-6 requirements. Am J Clin Nutr.

[15] Kennedy DO, Stevenson EJ, Jackson PA, Dunn S, Wishart K, Bieri G (2016). Multivitamins and minerals modulate whole-body energy metabolism and cerebral blood-flow during cognitive task performance: a double-blind, randomised, placebo-controlled trial. Nutr Metab (Lond).

[16] Haskell CF, Robertson B, Jones E, Forster J, Jones R, Wilde A (2010). Effects of a multi-vitamin/mineral supplement on cognitive function and fatigue during extended multi-tasking. Hum Psychopharmacol Clin Exp.

[17] Long SJ, Benton D (2013). Effects of vitamin and mineral supplementation on stress, mild psychiatric symptoms, and mood in nonclinical samples: a meta-analysis. Psychosom Med.

[18] Kennedy DO, Veasey R, Watson A, Dodd F, Jones E, Maggini S (2010). Effects of high-dose B vitamin complex with vitamin C and minerals on subjective mood and performance in healthy males. Psychopharmacology.

[19] Herrmann M, Schorr H, Obeid R, Scharhag J, Urhausen A, Kindermann W (2003). Homocysteine increases during endurance exercise. Clin Chem Lab Med.

[20] Herrmann M, Wilkinson J, Schorr H, Obeid R, Georg T, Urhausen A (2003). Comparison of the influence of volume-oriented training and high-intensity interval training on serum homocysteine and its cofactors in young, healthy swimmers. Clin Chem Lab Med.

[21] Nygård O, Vollset SE, Refsum H, Stensvold I, Tverdal A, Nordrehaug JE (1995). Total plasma homocysteine and cardiovascular risk profile: the Hordaland Homocysteine study. JAMA.

[22] Veasey RC, Haskell-Ramsay CF, Kennedy DO, Wishart K, Maggini S, Fuchs CJ (2015). The effects of supplementation with a vitamin and mineral complex with Guarana prior to fasted exercise on affect, exertion, cognitive performance, and substrate metabolism: a randomized controlled trial. Nutrient.

[23] Frayn K (1983). Calculation of substrate oxidation rates in vivo from gaseous exchange. J Appl Physiol.

[24] Borg GA (1973). Perceived exertion: a note on "history" and methods. Med Sci Sports.

[25] Dodd F, Kennedy D, Riby L, Haskell-Ramsay C (2015). A double-blind, placebo-controlled study evaluating the effects of caffeine and L-theanine both alone and in combination on cerebral blood flow, cognition and mood. Psychopharmacology.

[26] Haskell-Ramsay C, Jackson P, Forster J, Dodd F, Bowerbank S, Kennedy D (2018). The acute effects of caffeinated black coffee on cognition and mood in healthy young and older adults. Nutrients.

[27] Stonehouse W, Conlon CA, Podd J, Hill SR, Minihane AM, Haskell C (2013). DHA supplementation improved both memory and reaction time in healthy young adults: a randomized controlled trial. Am Clin Nutr.

[28] Kennedy D, Haskell C, Robertson B, Reay J, Brewster-Maund C, Luedemann J (2008). Improved cognitive performance and mental fatigue following a multi-vitamin and mineral supplement with added guarana (Paullinia cupana). Appetite.

[29] Scholey AB, French SJ, Morris PJ, Kennedy DO, Milne AL, Haskell CF (2010). Consumption of cocoa flavanols results in acute improvements in mood and cognitive performance during sustained mental effort. J Psychopharmacol.

[30] Craig CL, Marshall AL, Sjorstrom M, Bauman AE, Booth ML, Ainsworth BE (2003). International physical activity questionnaire: 12-country reliability and validity. Med Sci Sports Exerc.

[31] Williams C, Nute MG, Broadbank L, Vinall S (1990). Influence of fluid intake on endurance running performance - a COMPARISON between water, glucose and fructose solutions. Eur J Appl Physiol Occup Physiol.

[32] Spriet LL (2014). New insights into the interaction of carbohydrate and fat metabolism during exercise. Sports Med.

[33] Li Y, Wang C, Zhu K, Feng R, Sun C (2010). Effects of multivitamin and mineral supplementation on adiposity, energy expenditure and lipid profiles in obese Chinese women. Int J Obes.

[34] Achten J, Jeukendrup A (2003). Maximal fat oxidation during exercise in trained men. Int J Sports Med.

[35] Leckey JJ, Burke LM, Morton JP, Hawley JA (2015). Altering fatty acid availability does not impair prolonged, continuous running to fatigue: evidence for carbohydrate dependence. J Appl Physiol.

[36] Harris E, Kirk J, Rowsell R, Vitetta L, Sali A, Scholey AB (2011). The effect of multivitamin supplementation on mood and stress in healthy older men. Hum Psychopharmacol Clin Exp.

[37] Pipingas A, Camfield DA, Stough C, Cox KH, Fogg E, Tiplady B (2013). The effects of multivitamin supplementation on mood and general well-being in healthy young adults. A laboratory and at-home mobile phone assessment. Appetite.

[38] Jackson A, Stanforth P, Gagnon J, Rankinen T, Leon A, Rao D (2002). The effect of sex, age and race on estimating percentage body fat from body mass index: the heritage family study. Int J Obes.

[39] Venables MC, Achten J, Jeukendrup AE (2005). Determinants of fat oxidation during exercise in healthy men and women: a cross-sectional study. J Appl Physiol.

[40] Howe JC, Rumpler WV, Seale JL (1993). Energy expenditure by indirect calorimetry in premenopausal women: variation within one menstrual cycle. J Nutr Biochem.

[41] Oosthuyse T, Bosch AN (2010). The effect of the menstrual cycle on exercise metabolism. Sports Med.

[42] Albert PR (2015). Why is depression more prevalent in women?. J Psychiatry Neurosci.

[43] Bandelow B, Michaelis S (2015). Epidemiology of anxiety disorders in the 21st century. Dialogues Clin Neurosci.

[44] Kelly Megan M., Tyrka Audrey R., Anderson George M., Price Lawrence H., Carpenter Linda L. (2008). Sex differences in emotional and physiological responses to the Trier Social Stress Test. Journal of Behavior Therapy and Experimental Psychiatry.

[45] Beard John L., Hendricks Michael K., Perez Eva M., Murray-Kolb Laura E., Berg Astrid, Vernon-Feagans Lynne, Irlam James, Isaacs Washiefa, Sive Alan, Tomlinson Mark (2005). Maternal Iron Deficiency Anemia Affects Postpartum Emotions and Cognition. The Journal of Nutrition.

[46] Favrat Bernard, Balck Katharina, Breymann Christian, Hedenus Michael, Keller Thomas, Mezzacasa Anna, Gasche Christoph (2014). Evaluation of a Single Dose of Ferric Carboxymaltose in Fatigued, Iron-Deficient Women – PREFER a Randomized, Placebo-Controlled Study. PLoS ONE.

[47] Begdache L, Kianmehr H, Sabounchi N, Chaar M, Marhaba J. Principal component analysis identifies differential gender-specific dietary patterns that may be linked to mental distress in human adults. Nutr Neurosci. 2018;21:1–4. 10.1080/1028415X.2018.1500198 30028276

[48] Scholey A, Bauer I, Neale C, Savage K, Camfield D, White D (2013). Acute effects of different multivitamin mineral preparations with and without guaraná on mood, cognitive performance and functional brain activation. Nutrients.

[49] White DJ, Camfield DA, Maggini S, Pipingas A, Silberstein R, Stough C (2017). The effect of a single dose of multivitamin and mineral combinations with and without guaraná on functional brain activity during a continuous performance task. Nutr Neurosci.

[50] Lukaski HC (2004). Vitamin and mineral status: effects on physical performance. Nutrition.

[51] Pilchova I, Klacanova K, Tatarkova Z, Kaplan P, Racay P. The involvement of Mg2. Oxidative Med Cell Longev. 2017;2017:6797460. 10.1155/2017/6797460 PMC5516748 28757913

[52] Nelson M, Erens B, Bates B, Church S, Boshier T (2007). Low income diet and nutrition survey: TSO London.

[53] Troesch B, Hoeft B, McBurney M, Eggersdorfer M, Weber P (2012). Dietary surveys indicate vitamin intakes below recommendations are common in representative Western countries. Br J Nutr.

[54] Bruner AB, Joffe A, Duggan AK, Casella JF, Brandt J (1996). Randomised study of cognitive effects of iron supplementation in non-anaemic iron-deficient adolescent girls. Lancet.

