# Supplementary material for: Acute and chronic effects of multivitamin/mineral supplementation on objective and subjective energy measures
Source: Nutr Metab (Lond). 2020 Feb 24;17:16. doi: 10.1186/s12986-020-00435-1 (PMC7038616; doi:10.1186/s12986-020-00435-1)
Supplement: Supplementary file 1 — Additional file 1: Table S1. Indirect calorimetry during exercise. Table S2. Indirect calorimetry during cognitive tasks. Table S3. Tiredness ratings during exercise and cognitive tasks. Table S4. Rating of Perceived Exertion during exercise. Table S5. Energy and stress ratings during cognitive tasks. Table S6. Cognitive task performance. Table S7. Micronutrient, creatinine and homocysteine levels. Table S8. Recovery biomarker levels. [file 12986_2020_435_MOESM1_ESM.docx]

Supplementary table 1 – Indirect calorimetry during exercise

| Unadjusted means ± SD | | |  | **Baseline** | |  |  | **Day 1** | | | **Day 28** | |
| --- | --- | --- | --- | --- | --- | --- | --- | --- | --- | --- | --- | --- |
|  |  |  | N | Mean | SD | Repetition | Mean | | SD | Mean | | SD |
| Respiratory Exchange Rate | PLA | Male | 24 | 0.85 | 0.06 | Pre | 0.80 | | 0.05 | 0.81 | | 0.07 |
|  |  |  |  |  |  | 10 mins | 0.85 | | 0.04 | 0.87 | | 0.03 |
|  |  |  |  |  |  | 20 mins | 0.89 | | 0.04 | 0.91 | | 0.04 |
|  |  |  |  |  |  | 30 mins | 0.92 | | 0.05 | 0.94 | | 0.04 |
|  |  | Female | 14 | 0.86 | 0.06 | Pre | 0.81 | | 0.06 | 0.82 | | 0.06 |
|  |  |  |  |  |  | 10 mins | 0.84 | | 0.05 | 0.85 | | 0.04 |
|  |  |  |  |  |  | 20 mins | 0.86 | | 0.05 | 0.88 | | 0.05 |
|  |  |  |  |  |  | 30 mins | 0.88 | | 0.06 | 0.90 | | 0.06 |
|  | MVM | Male | 24 | 0.84 | 0.07 | Pre | 0.82 | | 0.07 | 0.83 | | 0.07 |
|  |  |  |  |  |  | 10 mins | 0.86 | | 0.07 | 0.87 | | 0.05 |
|  |  |  |  |  |  | 20 mins | 0.89 | | 0.06 | 0.91 | | 0.05 |
|  |  |  |  |  |  | 30 mins | 0.92 | | 0.08 | 0.94 | | 0.05 |
|  |  | Female | 17 | 0.84 | 0.07 | Pre | 0.81 | | 0.07 | 0.82 | | 0.08 |
|  |  |  |  |  |  | 10 mins | 0.83 | | 0.05 | 0.83 | | 0.08 |
|  |  |  |  |  |  | 20 mins | 0.86 | | 0.06 | 0.87 | | 0.09 |
|  |  |  |  |  |  | 30 mins | 0.87 | | 0.05 | 0.88 | | 0.09 |
| Carbohydrate Oxidation | PLA | Male | 24 | 0.16 | 0.06 | Pre | 0.12 | | 0.06 | 0.15 | | 0.11 |
|  |  |  |  |  |  | 10 mins | 1.47 | | 0.49 | 1.58 | | 0.39 |
|  |  |  |  |  |  | 20 mins | 1.89 | | 0.56 | 2.11 | | 0.57 |
|  |  |  |  |  |  | 30 mins | 2.74 | | 0.87 | 3.04 | | 0.80 |
|  |  | Female | 14 | 0.12 | 0.05 | Pre | 0.10 | | 0.06 | 0.12 | | 0.06 |
|  |  |  |  |  |  | 10 mins | 0.95 | | 0.35 | 0.96 | | 0.27 |
|  |  |  |  |  |  | 20 mins | 1.14 | | 0.37 | 1.21 | | 0.33 |
|  |  |  |  |  |  | 30 mins | 1.61 | | 0.46 | 1.75 | | 0.53 |
|  | MVM | Male | 24 | 0.15 | 0.10 | Pre | 0.17 | | 0.11 | 0.20 | | 0.11 |
|  |  |  |  |  |  | 10 mins | 1.60 | | 0.92 | 1.74 | | 0.70 |
|  |  |  |  |  |  | 20 mins | 2.02 | | 0.96 | 2.26 | | 0.78 |
|  |  |  |  |  |  | 30 mins | 3.04 | | 1.49 | 3.40 | | 1.11 |
|  |  | Female | 17 | 0.12 | 0.06 | Pre | 0.11 | | 0.07 | 0.13 | | 0.11 |
|  |  |  |  |  |  | 10 mins | 0.80 | | 0.31 | 0.87 | | 0.57 |
|  |  |  |  |  |  | 20 mins | 1.07 | | 0.37 | 1.13 | | 0.67 |
|  |  |  |  |  |  | 30 mins | 1.48 | | 0.47 | 1.54 | | 0.92 |
| Fat Oxidation | PLA | Male | 24 | 0.06 | 0.03 | Pre | 0.10 | | 0.04 | 0.10 | | 0.04 |
|  |  |  |  |  |  | 10 mins | 0.49 | | 0.19 | 0.44 | | 0.15 |
|  |  |  |  |  |  | 20 mins | 0.43 | | 0.19 | 0.32 | | 0.16 |
|  |  |  |  |  |  | 30 mins | 0.37 | | 0.27 | 0.24 | | 0.22 |
|  |  | Female | 14 | 0.04 | 0.02 | Pre | 0.07 | | 0.04 | 0.06 | | 0.02 |
|  |  |  |  |  |  | 10 mins | 0.39 | | 0.17 | 0.36 | | 0.13 |
|  |  |  |  |  |  | 20 mins | 0.36 | | 0.17 | 0.32 | | 0.15 |
|  |  |  |  |  |  | 30 mins | 0.40 | | 0.24 | 0.31 | | 0.23 |
|  | MVM | Male | 24 | 0.07 | 0.04 | Pre | 0.10 | | 0.05 | 0.10 | | 0.05 |
|  |  |  |  |  |  | 10 mins | 0.54 | | 0.41 | 0.47 | | 0.17 |
|  |  |  |  |  |  | 20 mins | 0.47 | | 0.43 | 0.35 | | 0.17 |
|  |  |  |  |  |  | 30 mins | 0.41 | | 0.63 | 0.24 | | 0.25 |
|  |  | Female | 17 | 0.05 | 0.03 | Pre | 0.09 | | 0.05 | 0.08 | | 0.05 |
|  |  |  |  |  |  | 10 mins | 0.43 | | 0.17 | 0.41 | | 0.25 |
|  |  |  |  |  |  | 20 mins | 0.38 | | 0.19 | 0.37 | | 0.29 |
|  |  |  |  |  |  | 30 mins | 0.42 | | 0.22 | 0.40 | | 0.38 |
| Energy Expenditure | PLA | Male | 24 | 1.20 | 0.24 | Pre | 1.40 | | 0.34 | 1.48 | | 0.40 |
|  |  |  |  |  |  | 10 mins | 10.26 | | 1.90 | 10.27 | | 2.08 |
|  |  |  |  |  |  | 20 mins | 11.41 | | 2.25 | 11.33 | | 2.33 |
|  |  |  |  |  |  | 30 mins | 14.31 | | 2.87 | 14.30 | | 2.78 |
|  |  | Female | 14 | 0.84 | 0.16 | Pre | 1.08 | | 0.30 | 1.06 | | 0.18 |
|  |  |  |  |  |  | 10 mins | 7.30 | | 1.10 | 7.08 | | 0.92 |
|  |  |  |  |  |  | 20 mins | 7.82 | | 1.27 | 7.74 | | 1.09 |
|  |  |  |  |  |  | 30 mins | 9.99 | | 1.37 | 9.85 | | 1.19 |
|  | MVM | Male | 24 | 1.23 | 0.27 | Pre | 1.57 | | 0.38 | 1.65 | | 0.50 |
|  |  |  |  |  |  | 10 mins | 11.27 | | 2.59 | 11.21 | | 2.61 |
|  |  |  |  |  |  | 20 mins | 12.35 | | 2.97 | 12.19 | | 2.88 |
|  |  |  |  |  |  | 30 mins | 15.86 | | 3.24 | 15.76 | | 3.34 |
|  |  | Female | 17 | 0.95 | 0.19 | Pre | 1.22 | | 0.32 | 1.21 | | 0.22 |
|  |  |  |  |  |  | 10 mins | 7.04 | | 1.24 | 7.12 | | 0.96 |
|  |  |  |  |  |  | 20 mins | 7.73 | | 1.37 | 7.81 | | 1.12 |
|  |  |  |  |  |  | 30 mins | 9.69 | | 1.64 | 9.80 | | 1.57 |

MVM=Multivitamin-Mineral; PLA=Placebo

Supplementary table 2 – Indirect calorimetry during cognitive tasks

| Unadjusted means ± SD | | |  | **Baseline** | |  |  |  | **Day 1** | | | **Day 28** | | |
| --- | --- | --- | --- | --- | --- | --- | --- | --- | --- | --- | --- | --- | --- | --- |
|  |  |  | N | Mean | SD | Task | Repetition | Mean | | SD | Mean | | SD |  |
| Respiratory Exchange Rate | PLA | Male | 24 | 0.85 | 0.06 | Serial 3s | 1.0 | 0.77 | | 0.06 | 0.77 | | 0.06 |  |
|  |  |  |  |  |  |  | 2.0 | 0.76 | | 0.05 | 0.76 | | 0.07 |  |
|  |  |  |  |  |  |  | 3.0 | 0.75 | | 0.06 | 0.75 | | 0.07 |  |
|  |  |  |  |  |  | Serial 7s | 1.0 | 0.78 | | 0.06 | 0.77 | | 0.07 |  |
|  |  |  |  |  |  |  | 2.0 | 0.75 | | 0.05 | 0.76 | | 0.07 |  |
|  |  |  |  |  |  |  | 3.0 | 0.74 | | 0.05 | 0.75 | | 0.07 |  |
|  |  |  |  |  |  | RVIP | 1.0 | 0.77 | | 0.05 | 0.76 | | 0.07 |  |
|  |  |  |  |  |  |  | 2.0 | 0.74 | | 0.05 | 0.75 | | 0.08 |  |
|  |  |  |  |  |  |  | 3.0 | 0.74 | | 0.05 | 0.75 | | 0.07 |  |
|  |  | Female | 14 | 0.86 | 0.06 | Serial 3s | 1.0 | 0.81 | | 0.08 | 0.79 | | 0.04 |  |
|  |  |  |  |  |  |  | 2.0 | 0.76 | | 0.04 | 0.77 | | 0.05 |  |
|  |  |  |  |  |  |  | 3.0 | 0.76 | | 0.04 | 0.77 | | 0.06 |  |
|  |  |  |  |  |  | Serial 7s | 1.0 | 0.78 | | 0.06 | 0.77 | | 0.06 |  |
|  |  |  |  |  |  |  | 2.0 | 0.75 | | 0.05 | 0.77 | | 0.06 |  |
|  |  |  |  |  |  |  | 3.0 | 0.75 | | 0.04 | 0.77 | | 0.06 |  |
|  |  |  |  |  |  | RVIP | 1.0 | 0.75 | | 0.04 | 0.76 | | 0.05 |  |
|  |  |  |  |  |  |  | 2.0 | 0.73 | | 0.04 | 0.75 | | 0.06 |  |
|  |  |  |  |  |  |  | 3.0 | 0.74 | | 0.05 | 0.75 | | 0.05 |  |
|  | MVM | Male | 24 | 0.84 | 0.07 | Serial 3s | 1.0 | 0.78 | | 0.05 | 0.76 | | 0.04 |  |
|  |  |  |  |  |  |  | 2.0 | 0.77 | | 0.05 | 0.76 | | 0.05 |  |
|  |  |  |  |  |  |  | 3.0 | 0.75 | | 0.06 | 0.75 | | 0.05 |  |
|  |  |  |  |  |  | Serial 7s | 1.0 | 0.78 | | 0.04 | 0.76 | | 0.04 |  |
|  |  |  |  |  |  |  | 2.0 | 0.76 | | 0.04 | 0.76 | | 0.05 |  |
|  |  |  |  |  |  |  | 3.0 | 0.75 | | 0.04 | 0.75 | | 0.04 |  |
|  |  |  |  |  |  | RVIP | 1.0 | 0.77 | | 0.04 | 0.75 | | 0.05 |  |
|  |  |  |  |  |  |  | 2.0 | 0.75 | | 0.04 | 0.74 | | 0.05 |  |
|  |  |  |  |  |  |  | 3.0 | 0.75 | | 0.05 | 0.75 | | 0.05 |  |
|  |  | Female | 17 | 0.84 | 0.07 | Serial 3s | 1.0 | 0.79 | | 0.05 | 0.78 | | 0.05 |  |
|  |  |  |  |  |  |  | 2.0 | 0.75 | | 0.06 | 0.75 | | 0.04 |  |
|  |  |  |  |  |  |  | 3.0 | 0.73 | | 0.05 | 0.75 | | 0.05 |  |
|  |  |  |  |  |  | Serial 7s | 1.0 | 0.78 | | 0.05 | 0.77 | | 0.05 |  |
|  |  |  |  |  |  |  | 2.0 | 0.74 | | 0.04 | 0.75 | | 0.04 |  |
|  |  |  |  |  |  |  | 3.0 | 0.73 | | 0.04 | 0.75 | | 0.05 |  |
|  |  |  |  |  |  | RVIP | 1.0 | 0.74 | | 0.04 | 0.75 | | 0.04 |  |
|  |  |  |  |  |  |  | 2.0 | 0.72 | | 0.05 | 0.75 | | 0.05 |  |
|  |  |  |  |  |  |  | 3.0 | 0.72 | | 0.05 | 0.74 | | 0.05 |  |
| Carbohydrate Oxidation | PLA | Male | 24 | 0.16 | 0.06 | Serial 3s | 1.0 | 0.09 | | 0.09 | 0.08 | | 0.09 |  |
|  |  |  |  |  |  |  | 2.0 | 0.06 | | 0.07 | 0.06 | | 0.09 |  |
|  |  |  |  |  |  |  | 3.0 | 0.06 | | 0.06 | 0.05 | | 0.09 |  |
|  |  |  |  |  |  | Serial 7s | 1.0 | 0.10 | | 0.09 | 0.09 | | 0.10 |  |
|  |  |  |  |  |  |  | 2.0 | 0.05 | | 0.06 | 0.06 | | 0.09 |  |
|  |  |  |  |  |  |  | 3.0 | 0.04 | | 0.05 | 0.05 | | 0.09 |  |
|  |  |  |  |  |  | RVIP | 1.0 | 0.08 | | 0.07 | 0.07 | | 0.09 |  |
|  |  |  |  |  |  |  | 2.0 | 0.04 | | 0.05 | 0.05 | | 0.08 |  |
|  |  |  |  |  |  |  | 3.0 | 0.05 | | 0.05 | 0.05 | | 0.08 |  |
|  |  | Female | 14 | 0.12 | 0.05 | Serial 3s | 1.0 | 0.11 | | 0.09 | 0.09 | | 0.04 |  |
|  |  |  |  |  |  |  | 2.0 | 0.06 | | 0.04 | 0.06 | | 0.04 |  |
|  |  |  |  |  |  |  | 3.0 | 0.06 | | 0.04 | 0.07 | | 0.05 |  |
|  |  |  |  |  |  | Serial 7s | 1.0 | 0.08 | | 0.07 | 0.06 | | 0.05 |  |
|  |  |  |  |  |  |  | 2.0 | 0.05 | | 0.05 | 0.07 | | 0.06 |  |
|  |  |  |  |  |  |  | 3.0 | 0.05 | | 0.05 | 0.06 | | 0.05 |  |
|  |  |  |  |  |  | RVIP | 1.0 | 0.04 | | 0.04 | 0.05 | | 0.05 |  |
|  |  |  |  |  |  |  | 2.0 | 0.02 | | 0.04 | 0.04 | | 0.05 |  |
|  |  |  |  |  |  |  | 3.0 | 0.03 | | 0.05 | 0.04 | | 0.04 |  |
|  | MVM | Male | 24 | 0.15 | 0.10 | Serial 3s | 1.0 | 0.11 | | 0.09 | 0.09 | | 0.06 |  |
|  |  |  |  |  |  |  | 2.0 | 0.09 | | 0.08 | 0.07 | | 0.06 |  |
|  |  |  |  |  |  |  | 3.0 | 0.06 | | 0.08 | 0.06 | | 0.07 |  |
|  |  |  |  |  |  | Serial 7s | 1.0 | 0.10 | | 0.06 | 0.09 | | 0.07 |  |
|  |  |  |  |  |  |  | 2.0 | 0.07 | | 0.06 | 0.07 | | 0.07 |  |
|  |  |  |  |  |  |  | 3.0 | 0.06 | | 0.06 | 0.06 | | 0.05 |  |
|  |  |  |  |  |  | RVIP | 1.0 | 0.09 | | 0.07 | 0.07 | | 0.08 |  |
|  |  |  |  |  |  |  | 2.0 | 0.06 | | 0.05 | 0.05 | | 0.07 |  |
|  |  |  |  |  |  |  | 3.0 | 0.06 | | 0.07 | 0.06 | | 0.08 |  |
|  |  | Female | 17 | 0.12 | 0.06 | Serial 3s | 1.0 | 0.09 | | 0.06 | 0.08 | | 0.06 |  |
|  |  |  |  |  |  |  | 2.0 | 0.04 | | 0.05 | 0.05 | | 0.04 |  |
|  |  |  |  |  |  |  | 3.0 | 0.02 | | 0.05 | 0.04 | | 0.05 |  |
|  |  |  |  |  |  | Serial 7s | 1.0 | 0.08 | | 0.04 | 0.07 | | 0.06 |  |
|  |  |  |  |  |  |  | 2.0 | 0.04 | | 0.04 | 0.04 | | 0.04 |  |
|  |  |  |  |  |  |  | 3.0 | 0.03 | | 0.04 | 0.04 | | 0.05 |  |
|  |  |  |  |  |  | RVIP | 1.0 | 0.03 | | 0.04 | 0.04 | | 0.04 |  |
|  |  |  |  |  |  |  | 2.0 | 0.02 | | 0.04 | 0.04 | | 0.05 |  |
|  |  |  |  |  |  |  | 3.0 | 0.02 | | 0.04 | 0.03 | | 0.05 |  |
| Fat Oxidation | PLA | Male | 24 | 0.06 | 0.03 | Serial 3s | 1.0 | 0.12 | | 0.04 | 0.12 | | 0.05 |  |
|  |  |  |  |  |  |  | 2.0 | 0.12 | | 0.05 | 0.13 | | 0.06 |  |
|  |  |  |  |  |  |  | 3.0 | 0.12 | | 0.04 | 0.14 | | 0.06 |  |
|  |  |  |  |  |  | Serial 7s | 1.0 | 0.12 | | 0.04 | 0.12 | | 0.05 |  |
|  |  |  |  |  |  |  | 2.0 | 0.12 | | 0.04 | 0.13 | | 0.05 |  |
|  |  |  |  |  |  |  | 3.0 | 0.13 | | 0.04 | 0.13 | | 0.06 |  |
|  |  |  |  |  |  | RVIP | 1.0 | 0.11 | | 0.03 | 0.11 | | 0.05 |  |
|  |  |  |  |  |  |  | 2.0 | 0.12 | | 0.04 | 0.12 | | 0.05 |  |
|  |  |  |  |  |  |  | 3.0 | 0.12 | | 0.04 | 0.12 | | 0.05 |  |
|  |  | Female | 14 | 0.04 | 0.02 | Serial 3s | 1.0 | 0.07 | | 0.03 | 0.08 | | 0.02 |  |
|  |  |  |  |  |  |  | 2.0 | 0.09 | | 0.02 | 0.08 | | 0.02 |  |
|  |  |  |  |  |  |  | 3.0 | 0.09 | | 0.02 | 0.08 | | 0.02 |  |
|  |  |  |  |  |  | Serial 7s | 1.0 | 0.08 | | 0.03 | 0.09 | | 0.03 |  |
|  |  |  |  |  |  |  | 2.0 | 0.09 | | 0.02 | 0.08 | | 0.03 |  |
|  |  |  |  |  |  |  | 3.0 | 0.10 | | 0.02 | 0.08 | | 0.02 |  |
|  |  |  |  |  |  | RVIP | 1.0 | 0.09 | | 0.02 | 0.08 | | 0.02 |  |
|  |  |  |  |  |  |  | 2.0 | 0.09 | | 0.02 | 0.08 | | 0.02 |  |
|  |  |  |  |  |  |  | 3.0 | 0.09 | | 0.02 | 0.08 | | 0.02 |  |
|  | MVM | Male | 24 | 0.07 | 0.04 | Serial 3s | 1.0 | 0.13 | | 0.05 | 0.13 | | 0.03 |  |
|  |  |  |  |  |  |  | 2.0 | 0.12 | | 0.05 | 0.13 | | 0.05 |  |
|  |  |  |  |  |  |  | 3.0 | 0.14 | | 0.06 | 0.13 | | 0.05 |  |
|  |  |  |  |  |  | Serial 7s | 1.0 | 0.12 | | 0.04 | 0.13 | | 0.04 |  |
|  |  |  |  |  |  |  | 2.0 | 0.13 | | 0.04 | 0.13 | | 0.04 |  |
|  |  |  |  |  |  |  | 3.0 | 0.14 | | 0.05 | 0.13 | | 0.03 |  |
|  |  |  |  |  |  | RVIP | 1.0 | 0.12 | | 0.03 | 0.13 | | 0.04 |  |
|  |  |  |  |  |  |  | 2.0 | 0.13 | | 0.04 | 0.13 | | 0.04 |  |
|  |  |  |  |  |  |  | 3.0 | 0.13 | | 0.04 | 0.13 | | 0.04 |  |
|  |  | Female | 17 | 0.05 | 0.03 | Serial 3s | 1.0 | 0.08 | | 0.03 | 0.09 | | 0.04 |  |
|  |  |  |  |  |  |  | 2.0 | 0.10 | | 0.04 | 0.10 | | 0.04 |  |
|  |  |  |  |  |  |  | 3.0 | 0.10 | | 0.04 | 0.10 | | 0.04 |  |
|  |  |  |  |  |  | Serial 7s | 1.0 | 0.09 | | 0.03 | 0.09 | | 0.04 |  |
|  |  |  |  |  |  |  | 2.0 | 0.10 | | 0.03 | 0.11 | | 0.04 |  |
|  |  |  |  |  |  |  | 3.0 | 0.10 | | 0.03 | 0.11 | | 0.04 |  |
|  |  |  |  |  |  | RVIP | 1.0 | 0.09 | | 0.03 | 0.10 | | 0.03 |  |
|  |  |  |  |  |  |  | 2.0 | 0.10 | | 0.03 | 0.10 | | 0.04 |  |
|  |  |  |  |  |  |  | 3.0 | 0.10 | | 0.04 | 0.11 | | 0.03 |  |
| Energy Expenditure | PLA | Male | 24 | 1.20 | 0.24 | Serial 3s | 1.0 | 1.44 | | 0.30 | 1.45 | | 0.28 |  |
|  |  |  |  |  |  |  | 2.0 | 1.34 | | 0.29 | 1.44 | | 0.26 |  |
|  |  |  |  |  |  |  | 3.0 | 1.36 | | 0.25 | 1.42 | | 0.30 |  |
|  |  |  |  |  |  | Serial 7s | 1.0 | 1.45 | | 0.30 | 1.43 | | 0.27 |  |
|  |  |  |  |  |  |  | 2.0 | 1.30 | | 0.25 | 1.43 | | 0.27 |  |
|  |  |  |  |  |  |  | 3.0 | 1.31 | | 0.24 | 1.41 | | 0.30 |  |
|  |  |  |  |  |  | RVIP | 1.0 | 1.28 | | 0.27 | 1.30 | | 0.24 |  |
|  |  |  |  |  |  |  | 2.0 | 1.22 | | 0.26 | 1.31 | | 0.25 |  |
|  |  |  |  |  |  |  | 3.0 | 1.28 | | 0.34 | 1.31 | | 0.26 |  |
|  |  | Female | 14 | 0.84 | 0.16 | Serial 3s | 1.0 | 1.10 | | 0.14 | 1.03 | | 0.12 |  |
|  |  |  |  |  |  |  | 2.0 | 1.01 | | 0.11 | 0.99 | | 0.11 |  |
|  |  |  |  |  |  |  | 3.0 | 1.02 | | 0.13 | 0.98 | | 0.11 |  |
|  |  |  |  |  |  | Serial 7s | 1.0 | 1.04 | | 0.17 | 1.05 | | 0.11 |  |
|  |  |  |  |  |  |  | 2.0 | 1.01 | | 0.13 | 1.02 | | 0.14 |  |
|  |  |  |  |  |  |  | 3.0 | 1.04 | | 0.13 | 0.94 | | 0.11 |  |
|  |  |  |  |  |  | RVIP | 1.0 | 0.93 | | 0.15 | 0.93 | | 0.16 |  |
|  |  |  |  |  |  |  | 2.0 | 0.92 | | 0.12 | 0.91 | | 0.12 |  |
|  |  |  |  |  |  |  | 3.0 | 0.92 | | 0.13 | 0.89 | | 0.12 |  |
|  | MVM | Male | 24 | 1.23 | 0.27 | Serial 3s | 1.0 | 1.57 | | 0.33 | 1.53 | | 0.30 |  |
|  |  |  |  |  |  |  | 2.0 | 1.45 | | 0.37 | 1.46 | | 0.28 |  |
|  |  |  |  |  |  |  | 3.0 | 1.52 | | 0.44 | 1.45 | | 0.25 |  |
|  |  |  |  |  |  | Serial 7s | 1.0 | 1.52 | | 0.29 | 1.52 | | 0.26 |  |
|  |  |  |  |  |  |  | 2.0 | 1.50 | | 0.29 | 1.46 | | 0.25 |  |
|  |  |  |  |  |  |  | 3.0 | 1.48 | | 0.33 | 1.42 | | 0.23 |  |
|  |  |  |  |  |  | RVIP | 1.0 | 1.48 | | 0.28 | 1.41 | | 0.26 |  |
|  |  |  |  |  |  |  | 2.0 | 1.43 | | 0.34 | 1.37 | | 0.24 |  |
|  |  |  |  |  |  |  | 3.0 | 1.43 | | 0.30 | 1.37 | | 0.25 |  |
|  |  | Female | 17 | 0.95 | 0.19 | Serial 3s | 1.0 | 1.12 | | 0.23 | 1.14 | | 0.22 |  |
|  |  |  |  |  |  |  | 2.0 | 1.04 | | 0.21 | 1.09 | | 0.23 |  |
|  |  |  |  |  |  |  | 3.0 | 1.02 | | 0.28 | 1.10 | | 0.25 |  |
|  |  |  |  |  |  | Serial 7s | 1.0 | 1.10 | | 0.23 | 1.14 | | 0.20 |  |
|  |  |  |  |  |  |  | 2.0 | 1.06 | | 0.22 | 1.12 | | 0.23 |  |
|  |  |  |  |  |  |  | 3.0 | 1.05 | | 0.23 | 1.12 | | 0.30 |  |
|  |  |  |  |  |  | RVIP | 1.0 | 0.98 | | 0.20 | 1.07 | | 0.20 |  |
|  |  |  |  |  |  |  | 2.0 | 0.99 | | 0.22 | 1.07 | | 0.17 |  |
|  |  |  |  |  |  |  | 3.0 | 1.01 | | 0.27 | 1.07 | | 0.20 |  |

MVM=Multivitamin-Mineral; PLA=Placebo; RVIP=Rapid Visual Information Processing

Supplementary table 3 – Tiredness ratings during exercise and cognitive tasks

|  |  |  |  |  |  |  |  | **Exercise** | | | | | | **Cognitive tasks** | | | | | |  | | |
| --- | --- | --- | --- | --- | --- | --- | --- | --- | --- | --- | --- | --- | --- | --- | --- | --- | --- | --- | --- | --- | --- | --- |
| Unadjusted means ± SD | | |  | **Baseline** | |  |  | **Day 1** | | | **Day 28** | | | | **Day 1** | | | **Day 28** | | | |  |
|  | | | N | Mean | SD | Repetition | Mean | | SD | Mean | | SD | Mean | | | SD | Mean | | SD | |  |  |
| Mental Tiredness | PLA | Male | 25 | 32.84 | 18.28 | Pre | 28.80 | | 13.81 | 23.33 | | 16.71 | 37.64 | | | 18.40 | 33.64 | | 17.92 | |  |  |
|  |  |  |  |  |  | 10 mins | 32.92 | | 10.95 | 27.56 | | 16.23 | 46.28 | | | 20.91 | 42.00 | | 18.03 | |  |  |
|  |  |  |  |  |  | 20 mins | 33.72 | | 13.43 | 31.56 | | 14.38 | 50.60 | | | 22.09 | 53.60 | | 15.82 | |  |  |
|  |  |  |  |  |  | 30 mins | 42.42 | | 14.69 | 35.56 | | 17.27 | 60.52 | | | 19.06 | 61.20 | | 19.45 | |  |  |
|  |  | Female | 15 | 46.60 | 18.52 | Pre | 41.87 | | 18.19 | 38.07 | | 23.04 | 45.00 | | | 20.93 | 45.87 | | 22.06 | |  |  |
|  |  |  |  |  |  | 10 mins | 39.27 | | 19.49 | 34.20 | | 19.24 | 53.33 | | | 19.44 | 49.13 | | 24.72 | |  |  |
|  |  |  |  |  |  | 20 mins | 41.07 | | 17.59 | 36.87 | | 18.49 | 65.00 | | | 14.92 | 64.20 | | 16.26 | |  |  |
|  |  |  |  |  |  | 30 mins | 45.93 | | 18.32 | 45.07 | | 21.57 | 64.73 | | | 19.38 | 68.40 | | 14.18 | |  |  |
|  | MVM | Male | 25 | 29.56 | 19.80 | Pre | 25.44 | | 14.73 | 21.12 | | 13.57 | 30.80 | | | 14.91 | 30.92 | | 19.67 | |  |  |
|  |  |  |  |  |  | 10 mins | 25.64 | | 12.45 | 20.44 | | 15.35 | 45.32 | | | 20.93 | 45.92 | | 20.29 | |  |  |
|  |  |  |  |  |  | 20 mins | 27.64 | | 9.95 | 23.96 | | 13.77 | 55.40 | | | 21.33 | 51.80 | | 22.43 | |  |  |
|  |  |  |  |  |  | 30 mins | 37.50 | | 13.62 | 33.80 | | 15.94 | 55.88 | | | 22.22 | 52.48 | | 22.93 | |  |  |
|  |  | Female | 17 | 43.24 | 18.84 | Pre | 32.00 | | 15.31 | 30.06 | | 17.01 | 40.24 | | | 20.93 | 38.00 | | 20.51 | |  |  |
|  |  |  |  |  |  | 10 mins | 27.35 | | 18.11 | 24.44 | | 9.49 | 48.41 | | | 21.52 | 51.75 | | 18.43 | |  |  |
|  |  |  |  |  |  | 20 mins | 33.18 | | 18.26 | 25.69 | | 10.03 | 56.76 | | | 23.27 | 54.56 | | 20.28 | |  |  |
|  |  |  |  |  |  | 30 mins | 36.76 | | 15.53 | 27.88 | | 8.79 | 62.41 | | | 19.48 | 58.56 | | 21.87 | |  |  |
| Physical Tiredness | PLA | Male | 25 | 32.84 | 19.15 | Pre | 28.16 | | 19.18 | 22.17 | | 18.83 | 38.60 | | | 15.49 | 37.24 | | 17.19 | |  |  |
|  |  |  |  |  |  | 10 mins | 36.80 | | 12.89 | 29.40 | | 17.59 | 42.00 | | | 15.36 | 41.00 | | 19.05 | |  |  |
|  |  |  |  |  |  | 20 mins | 43.96 | | 14.46 | 35.04 | | 16.23 | 45.52 | | | 16.09 | 44.56 | | 17.82 | |  |  |
|  |  |  |  |  |  | 30 mins | 60.75 | | 13.10 | 52.40 | | 11.51 | 44.72 | | | 19.52 | 47.60 | | 19.04 | |  |  |
|  |  | Female | 15 | 37.40 | 20.57 | Pre | 36.40 | | 21.12 | 29.20 | | 21.13 | 38.93 | | | 14.94 | 37.07 | | 18.21 | |  |  |
|  |  |  |  |  |  | 10 mins | 37.93 | | 16.26 | 37.47 | | 18.69 | 40.40 | | | 14.18 | 36.73 | | 19.24 | |  |  |
|  |  |  |  |  |  | 20 mins | 43.13 | | 15.89 | 43.13 | | 18.86 | 43.53 | | | 18.17 | 40.13 | | 20.96 | |  |  |
|  |  |  |  |  |  | 30 mins | 58.00 | | 18.33 | 63.36 | | 16.02 | 47.33 | | | 18.42 | 37.80 | | 22.40 | |  |  |
|  | MVM | Male | 25 | 26.48 | 19.75 | Pre | 24.76 | | 17.97 | 19.84 | | 16.45 | 39.64 | | | 18.57 | 35.72 | | 19.56 | |  |  |
|  |  |  |  |  |  | 10 mins | 32.12 | | 16.81 | 24.68 | | 14.44 | 37.48 | | | 19.79 | 35.12 | | 20.77 | |  |  |
|  |  |  |  |  |  | 20 mins | 36.76 | | 18.69 | 31.76 | | 13.31 | 38.40 | | | 21.49 | 35.24 | | 19.90 | |  |  |
|  |  |  |  |  |  | 30 mins | 59.58 | | 18.93 | 54.56 | | 16.60 | 35.28 | | | 23.46 | 34.80 | | 20.51 | |  |  |
|  |  | Female | 17 | 38.47 | 16.98 | Pre | 31.29 | | 18.24 | 27.50 | | 15.50 | 36.65 | | | 19.38 | 38.94 | | 17.19 | |  |  |
|  |  |  |  |  |  | 10 mins | 34.59 | | 14.08 | 31.38 | | 12.07 | 41.76 | | | 21.41 | 36.13 | | 17.46 | |  |  |
|  |  |  |  |  |  | 20 mins | 38.53 | | 15.61 | 34.94 | | 10.49 | 36.35 | | | 18.99 | 40.56 | | 19.77 | |  |  |
|  |  |  |  |  |  | 30 mins | 52.41 | | 15.47 | 51.94 | | 11.33 | 36.29 | | | 18.94 | 42.50 | | 19.22 | |  |  |

MVM=Multivitamin-Mineral; PLA=Placebo

Supplementary table 4 – Rating of Perceived Exertion during exercise

| Unadjusted means ± SD | | |  |  | **Day 1** | |  | **Day 28** | | |
| --- | --- | --- | --- | --- | --- | --- | --- | --- | --- | --- |
|  | | | Repetition | N | Mean | SD | Mean | | SD |  |
| RPE | PLA | Male | 10 mins | 25 | 9.00 | 2.00 | 9.00 | | 2.00 |  |
|  |  |  | 20 mins |  | 11.00 | 2.00 | 11.00 | | 2.00 |  |
|  |  |  | 30 mins |  | 15.00 | 2.00 | 14.00 | | 3.00 |  |
|  |  | Female | 10 mins | 17 | 10.00 | 2.00 | 10.00 | | 1.00 |  |
|  |  |  | 20 mins |  | 11.00 | 2.00 | 11.00 | | 1.00 |  |
|  |  |  | 30 mins |  | 15.00 | 1.00 | 15.00 | | 2.00 |  |
|  | MVM | Male | 10 mins | 25 | 10.00 | 2.00 | 10.00 | | 1.00 |  |
|  |  |  | 20 mins |  | 12.00 | 2.00 | 12.00 | | 1.00 |  |
|  |  |  | 30 mins |  | 15.00 | 2.00 | 14.00 | | 2.00 |  |
|  |  | Female | 10 mins | 15 | 10.00 | 2.00 | 11.00 | | 2.00 |  |
|  |  |  | 20 mins |  | 12.00 | 2.00 | 12.00 | | 2.00 |  |
|  |  |  | 30 mins |  | 16.00 | 1.00 | 15.00 | | 2.00 |  |

MVM=Multivitamin-Mineral; PLA=Placebo; RPE=Rating of Perceived Exertion

Supplementary table 5 – Energy and stress ratings during cognitive tasks

| Unadjusted means ± SD | | |  | **Baseline** | |  |  | **Day 1** | | | **Day 28** | | |
| --- | --- | --- | --- | --- | --- | --- | --- | --- | --- | --- | --- | --- | --- |
|  | | | N | Mean | SD | Repetition | Mean | | SD | Mean | | SD |  |
| Concentration | PLA | Male | 25 | 56.20 | 19.81 | Pre | 60.80 | | 15.37 | 60.36 | | 16.96 |  |
|  |  |  |  |  |  | 10 mins | 50.76 | | 23.15 | 52.76 | | 20.70 |  |
|  |  |  |  |  |  | 20 mins | 46.40 | | 24.25 | 44.08 | | 19.38 |  |
|  |  |  |  |  |  | 30 mins | 39.08 | | 25.44 | 41.72 | | 18.09 |  |
|  |  | Female | 15 | 51.73 | 18.62 | Pre | 54.67 | | 17.52 | 54.93 | | 23.32 |  |
|  |  |  |  |  |  | 10 mins | 46.33 | | 16.83 | 46.53 | | 23.02 |  |
|  |  |  |  |  |  | 20 mins | 35.60 | | 20.56 | 35.87 | | 15.22 |  |
|  |  |  |  |  |  | 30 mins | 30.40 | | 18.75 | 31.53 | | 17.40 |  |
|  | MVM | Male | 25 | 60.76 | 17.08 | Pre | 67.60 | | 13.60 | 66.04 | | 19.11 |  |
|  |  |  |  |  |  | 10 mins | 57.52 | | 19.68 | 56.88 | | 18.98 |  |
|  |  |  |  |  |  | 20 mins | 51.68 | | 22.53 | 50.84 | | 21.16 |  |
|  |  |  |  |  |  | 30 mins | 43.96 | | 25.04 | 50.68 | | 23.59 |  |
|  |  | Female | 17 | 55.94 | 18.43 | Pre | 58.94 | | 18.64 | 60.50 | | 16.80 |  |
|  |  |  |  |  |  | 10 mins | 55.12 | | 20.90 | 42.56 | | 20.43 |  |
|  |  |  |  |  |  | 20 mins | 46.76 | | 25.82 | 33.13 | | 20.42 |  |
|  |  |  |  |  |  | 30 mins | 42.18 | | 27.76 | 35.69 | | 26.08 |  |
| Mental Stamina | PLA | Male | 25 | 61.76 | 17.99 | Pre | 60.56 | | 13.31 | 64.60 | | 17.63 |  |
|  |  |  |  |  |  | 10 mins | 50.96 | | 17.60 | 55.08 | | 17.11 |  |
|  |  |  |  |  |  | 20 mins | 42.72 | | 19.84 | 44.76 | | 17.79 |  |
|  |  |  |  |  |  | 30 mins | 37.92 | | 20.25 | 42.44 | | 17.69 |  |
|  |  | Female | 15 | 52.27 | 17.93 | Pre | 52.27 | | 15.58 | 53.87 | | 22.83 |  |
|  |  |  |  |  |  | 10 mins | 43.73 | | 17.51 | 48.20 | | 24.28 |  |
|  |  |  |  |  |  | 20 mins | 37.60 | | 20.62 | 34.93 | | 19.68 |  |
|  |  |  |  |  |  | 30 mins | 35.53 | | 19.38 | 32.53 | | 15.91 |  |
|  | MVM | Male | 25 | 67.92 | 16.49 | Pre | 66.48 | | 14.67 | 68.48 | | 17.07 |  |
|  |  |  |  |  |  | 10 mins | 53.84 | | 18.01 | 56.00 | | 18.14 |  |
|  |  |  |  |  |  | 20 mins | 47.56 | | 22.50 | 46.56 | | 21.83 |  |
|  |  |  |  |  |  | 30 mins | 41.16 | | 24.95 | 46.12 | | 21.02 |  |
|  |  | Female | 17 | 58.35 | 15.85 | Pre | 58.76 | | 18.40 | 63.56 | | 13.70 |  |
|  |  |  |  |  |  | 10 mins | 51.18 | | 22.25 | 48.19 | | 18.97 |  |
|  |  |  |  |  |  | 20 mins | 45.24 | | 22.73 | 38.75 | | 21.86 |  |
|  |  |  |  |  |  | 30 mins | 40.94 | | 23.11 | 36.25 | | 22.92 |  |
| Physical Stamina | PLA | Male | 25 | 65.48 | 13.96 | Pre | 60.28 | | 15.76 | 61.16 | | 12.66 |  |
|  |  |  |  |  |  | 10 mins | 58.76 | | 13.51 | 59.72 | | 13.92 |  |
|  |  |  |  |  |  | 20 mins | 56.80 | | 14.65 | 54.52 | | 13.95 |  |
|  |  |  |  |  |  | 30 mins | 55.56 | | 17.64 | 56.40 | | 16.54 |  |
|  |  | Female | 15 | 54.07 | 17.94 | Pre | 54.80 | | 12.04 | 61.00 | | 15.48 |  |
|  |  |  |  |  |  | 10 mins | 52.40 | | 10.20 | 57.87 | | 14.66 |  |
|  |  |  |  |  |  | 20 mins | 49.27 | | 13.03 | 57.80 | | 17.02 |  |
|  |  |  |  |  |  | 30 mins | 52.47 | | 10.43 | 54.87 | | 15.98 |  |
|  | MVM | Male | 25 | 69.76 | 19.14 | Pre | 60.68 | | 15.45 | 61.84 | | 17.93 |  |
|  |  |  |  |  |  | 10 mins | 60.92 | | 17.13 | 63.32 | | 17.22 |  |
|  |  |  |  |  |  | 20 mins | 55.08 | | 21.07 | 63.96 | | 16.37 |  |
|  |  |  |  |  |  | 30 mins | 58.04 | | 19.74 | 60.08 | | 20.60 |  |
|  |  | Female | 17 | 62.65 | 13.18 | Pre | 59.65 | | 17.77 | 60.06 | | 15.77 |  |
|  |  |  |  |  |  | 10 mins | 59.06 | | 18.61 | 59.31 | | 16.14 |  |
|  |  |  |  |  |  | 20 mins | 56.41 | | 18.96 | 56.88 | | 15.38 |  |
|  |  |  |  |  |  | 30 mins | 59.82 | | 16.20 | 55.88 | | 13.02 |  |
| Stress | PLA | Male | 25 | 20.32 | 17.03 | Pre | 23.32 | | 17.88 | 21.04 | | 17.29 |  |
|  |  |  |  |  |  | 10 mins | 29.08 | | 20.20 | 23.76 | | 16.55 |  |
|  |  |  |  |  |  | 20 mins | 29.44 | | 17.68 | 29.88 | | 20.08 |  |
|  |  |  |  |  |  | 30 mins | 26.80 | | 18.75 | 31.36 | | 20.37 |  |
|  |  | Female | 15 | 29.27 | 19.85 | Pre | 30.87 | | 20.95 | 29.00 | | 28.85 |  |
|  |  |  |  |  |  | 10 mins | 38.67 | | 25.51 | 38.00 | | 29.53 |  |
|  |  |  |  |  |  | 20 mins | 39.87 | | 25.87 | 37.87 | | 32.05 |  |
|  |  |  |  |  |  | 30 mins | 38.20 | | 27.99 | 37.27 | | 32.66 |  |
|  | MVM | Male | 25 | 15.24 | 10.41 | Pre | 15.64 | | 11.11 | 16.28 | | 13.16 |  |
|  |  |  |  |  |  | 10 mins | 23.72 | | 12.54 | 21.76 | | 14.65 |  |
|  |  |  |  |  |  | 20 mins | 30.36 | | 20.83 | 25.20 | | 16.79 |  |
|  |  |  |  |  |  | 30 mins | 36.44 | | 26.42 | 21.96 | | 16.55 |  |
|  |  | Female | 17 | 30.24 | 21.55 | Pre | 27.47 | | 21.61 | 22.81 | | 16.11 |  |
|  |  |  |  |  |  | 10 mins | 29.47 | | 20.79 | 30.75 | | 16.72 |  |
|  |  |  |  |  |  | 20 mins | 27.41 | | 21.93 | 29.06 | | 15.67 |  |
|  |  |  |  |  |  | 30 mins | 26.12 | | 23.04 | 22.25 | | 15.27 |  |

MVM=Multivitamin-Mineral; PLA=Placebo

Supplementary table 6 – Cognitive task performance

| Unadjusted means ± SD | | | |  | **Day 1** | |  | **Day 28** | | |
| --- | --- | --- | --- | --- | --- | --- | --- | --- | --- | --- |
|  |  |  | Repetition | N | Mean | SD | Mean | | SD |  |
| Serial 3s Total | PLA | Male | 1 | 25 | 45.38 | 17.32 | 48.40 | | 16.50 |  |
|  |  |  | 2 |  | 46.92 | 15.63 | 50.36 | | 17.53 |  |
|  |  |  | 3 |  | 47.92 | 15.25 | 47.96 | | 15.18 |  |
|  |  | Female | 1 | 15 | 31.53 | 14.63 | 33.27 | | 11.99 |  |
|  |  |  | 2 |  | 32.93 | 11.74 | 36.27 | | 13.11 |  |
|  |  |  | 3 |  | 32.60 | 9.74 | 36.87 | | 9.49 |  |
|  | MVM | Male | 1 | 25 | 41.96 | 11.95 | 44.32 | | 12.26 |  |
|  |  |  | 2 |  | 44.64 | 13.23 | 46.56 | | 13.02 |  |
|  |  |  | 3 |  | 45.92 | 14.45 | 46.88 | | 14.26 |  |
|  |  | Female | 1 | 17 | 37.12 | 10.62 | 41.69 | | 10.64 |  |
|  |  |  | 2 |  | 40.82 | 10.93 | 43.63 | | 12.39 |  |
|  |  |  | 3 |  | 40.18 | 9.65 | 44.06 | | 11.24 |  |
| Serial 3s Correct | PLA | Male | 1 | 25 | 43.58 | 18.26 | 46.24 | | 17.29 |  |
|  |  |  | 2 |  | 45.16 | 15.53 | 48.16 | | 17.36 |  |
|  |  |  | 3 |  | 45.20 | 14.09 | 45.04 | | 15.04 |  |
|  |  | Female | 1 | 15 | 29.73 | 15.27 | 31.53 | | 12.57 |  |
|  |  |  | 2 |  | 30.20 | 12.20 | 33.80 | | 13.23 |  |
|  |  |  | 3 |  | 29.87 | 10.49 | 33.07 | | 11.05 |  |
|  | MVM | Male | 1 | 25 | 40.16 | 12.14 | 42.88 | | 12.94 |  |
|  |  |  | 2 |  | 42.40 | 13.26 | 45.04 | | 13.53 |  |
|  |  |  | 3 |  | 43.32 | 15.36 | 45.04 | | 14.11 |  |
|  |  | Female | 1 | 17 | 35.35 | 10.81 | 39.25 | | 11.87 |  |
|  |  |  | 2 |  | 39.18 | 11.50 | 40.56 | | 14.45 |  |
|  |  |  | 3 |  | 37.59 | 9.83 | 40.13 | | 13.58 |  |
| Serial 3s Errors | PLA | Male | 1 | 25 | 1.79 | 2.11 | 2.16 | | 2.43 |  |
|  |  |  | 2 |  | 1.76 | 1.59 | 2.20 | | 2.02 |  |
|  |  |  | 3 |  | 2.72 | 2.61 | 2.92 | | 2.53 |  |
|  |  | Female | 1 | 15 | 1.80 | 2.18 | 1.73 | | 2.34 |  |
|  |  |  | 2 |  | 2.73 | 3.56 | 2.47 | | 3.52 |  |
|  |  |  | 3 |  | 2.73 | 2.31 | 3.80 | | 5.32 |  |
|  | MVM | Male | 1 | 25 | 1.80 | 1.89 | 1.44 | | 2.12 |  |
|  |  |  | 2 |  | 2.24 | 1.64 | 1.52 | | 1.92 |  |
|  |  |  | 3 |  | 2.60 | 2.83 | 1.84 | | 1.89 |  |
|  |  | Female | 1 | 17 | 1.76 | 2.05 | 2.44 | | 4.11 |  |
|  |  |  | 2 |  | 1.65 | 1.54 | 3.06 | | 4.97 |  |
|  |  |  | 3 |  | 2.59 | 2.45 | 3.94 | | 5.04 |  |
| Serial 7s Total | PLA | Male | 1 | 25 | 28.88 | 11.16 | 29.32 | | 13.08 |  |
|  |  |  | 2 |  | 27.96 | 11.14 | 29.76 | | 14.11 |  |
|  |  |  | 3 |  | 29.84 | 12.08 | 30.84 | | 14.77 |  |
|  |  | Female | 1 | 15 | 18.67 | 6.41 | 19.53 | | 6.93 |  |
|  |  |  | 2 |  | 19.47 | 7.50 | 19.73 | | 7.55 |  |
|  |  |  | 3 |  | 20.93 | 9.18 | 21.53 | | 6.99 |  |
|  | MVM | Male | 1 | 25 | 27.24 | 9.76 | 27.80 | | 9.26 |  |
|  |  |  | 2 |  | 29.08 | 10.52 | 29.20 | | 10.93 |  |
|  |  |  | 3 |  | 29.24 | 10.24 | 30.56 | | 10.28 |  |
|  |  | Female | 1 | 17 | 21.12 | 6.50 | 22.88 | | 8.45 |  |
|  |  |  | 2 |  | 22.06 | 7.53 | 25.13 | | 7.63 |  |
|  |  |  | 3 |  | 24.12 | 7.98 | 25.06 | | 9.41 |  |
| Serial 7s Correct | PLA | Male | 1 | 25 | 27.08 | 11.38 | 27.08 | | 13.52 |  |
|  |  |  | 2 |  | 25.12 | 11.49 | 26.96 | | 14.50 |  |
|  |  |  | 3 |  | 27.36 | 12.21 | 28.00 | | 15.49 |  |
|  |  | Female | 1 | 15 | 15.47 | 7.84 | 16.33 | | 8.37 |  |
|  |  |  | 2 |  | 16.47 | 7.51 | 16.67 | | 7.83 |  |
|  |  |  | 3 |  | 17.87 | 9.77 | 18.33 | | 7.18 |  |
|  | MVM | Male | 1 | 25 | 25.48 | 10.37 | 25.60 | | 9.65 |  |
|  |  |  | 2 |  | 26.48 | 11.30 | 26.56 | | 11.32 |  |
|  |  |  | 3 |  | 26.24 | 10.34 | 27.84 | | 9.82 |  |
|  |  | Female | 1 | 17 | 18.47 | 7.35 | 20.13 | | 8.98 |  |
|  |  |  | 2 |  | 19.47 | 8.52 | 21.63 | | 9.11 |  |
|  |  |  | 3 |  | 21.76 | 8.08 | 22.44 | | 9.09 |  |
| Serial 7s Errors | PLA | Male | 1 | 25 | 1.80 | 1.50 | 2.24 | | 2.01 |  |
|  |  |  | 2 |  | 2.84 | 1.93 | 2.80 | | 1.80 |  |
|  |  |  | 3 |  | 2.48 | 2.52 | 2.84 | | 2.34 |  |
|  |  | Female | 1 | 15 | 3.20 | 3.21 | 3.20 | | 3.69 |  |
|  |  |  | 2 |  | 3.00 | 2.80 | 3.07 | | 2.89 |  |
|  |  |  | 3 |  | 3.07 | 2.05 | 3.20 | | 4.38 |  |
|  | MVM | Male | 1 | 25 | 1.76 | 1.90 | 2.20 | | 2.06 |  |
|  |  |  | 2 |  | 2.60 | 2.60 | 2.64 | | 2.00 |  |
|  |  |  | 3 |  | 3.00 | 2.48 | 2.72 | | 2.30 |  |
|  |  | Female | 1 | 17 | 2.65 | 2.00 | 2.75 | | 2.46 |  |
|  |  |  | 2 |  | 2.59 | 2.12 | 3.50 | | 2.53 |  |
|  |  |  | 3 |  | 2.35 | 1.06 | 2.63 | | 1.78 |  |
| RVIP Accuracy | PLA | Male | 1 | 25 | 62.20 | 20.98 | 62.00 | | 20.30 |  |
|  |  |  | 2 |  | 59.00 | 16.74 | 62.30 | | 18.21 |  |
|  |  |  | 3 |  | 58.50 | 19.49 | 62.30 | | 17.12 |  |
|  |  | Female | 1 | 15 | 47.50 | 11.30 | 52.83 | | 19.27 |  |
|  |  |  | 2 |  | 47.00 | 14.31 | 49.83 | | 19.65 |  |
|  |  |  | 3 |  | 49.00 | 17.77 | 47.83 | | 18.99 |  |
|  | MVM | Male | 1 | 25 | 60.20 | 20.14 | 64.50 | | 18.30 |  |
|  |  |  | 2 |  | 60.60 | 19.33 | 63.70 | | 19.51 |  |
|  |  |  | 3 |  | 61.60 | 18.73 | 64.60 | | 18.28 |  |
|  |  | Female | 1 | 17 | 62.94 | 15.72 | 63.44 | | 19.28 |  |
|  |  |  | 2 |  | 63.09 | 17.71 | 59.06 | | 23.02 |  |
|  |  |  | 3 |  | 60.44 | 19.67 | 59.38 | | 25.34 |  |
| RVIP RT | PLA | Male | 1 | 25 | 476.44 | 39.73 | 475.39 | | 32.95 |  |
|  |  |  | 2 |  | 486.59 | 33.13 | 486.37 | | 41.09 |  |
|  |  |  | 3 |  | 489.06 | 33.09 | 486.42 | | 34.08 |  |
|  |  | Female | 1 | 15 | 504.41 | 52.43 | 497.10 | | 37.80 |  |
|  |  |  | 2 |  | 507.50 | 46.79 | 493.75 | | 47.72 |  |
|  |  |  | 3 |  | 506.21 | 53.81 | 500.80 | | 56.63 |  |
|  | MVM | Male | 1 | 25 | 493.25 | 48.06 | 493.09 | | 43.72 |  |
|  |  |  | 2 |  | 491.22 | 55.35 | 490.51 | | 50.26 |  |
|  |  |  | 3 |  | 488.48 | 46.15 | 484.80 | | 44.48 |  |
|  |  | Female | 1 | 17 | 484.47 | 45.02 | 480.10 | | 42.29 |  |
|  |  |  | 2 |  | 498.55 | 48.28 | 493.64 | | 48.14 |  |
|  |  |  | 3 |  | 495.93 | 46.52 | 476.63 | | 50.18 |  |
| RVIP False Alarms | PLA | Male | 1 | 25 | 3.68 | 6.38 | 2.96 | | 4.68 |  |
|  |  |  | 2 |  | 1.96 | 2.99 | 2.60 | | 5.37 |  |
|  |  |  | 3 |  | 3.44 | 6.31 | 2.20 | | 3.10 |  |
|  |  | Female | 1 | 15 | 3.40 | 5.75 | 3.47 | | 7.09 |  |
|  |  |  | 2 |  | 2.60 | 3.25 | 3.13 | | 5.94 |  |
|  |  |  | 3 |  | 2.93 | 6.02 | 5.07 | | 10.28 |  |
|  | MVM | Male | 1 | 25 | 2.40 | 1.85 | 1.92 | | 1.73 |  |
|  |  |  | 2 |  | 2.48 | 2.22 | 1.72 | | 2.03 |  |
|  |  |  | 3 |  | 1.88 | 1.83 | 1.80 | | 1.55 |  |
|  |  | Female | 1 | 17 | 3.35 | 5.48 | 1.69 | | 1.40 |  |
|  |  |  | 2 |  | 2.35 | 2.52 | 2.13 | | 2.13 |  |
|  |  |  | 3 |  | 2.76 | 4.18 | 2.44 | | 2.90 |  |

MVM=Multivitamin-Mineral; PLA=Placebo; RT=Reaction Time; RVIP= Rapid Visual Information Processing

Supplementary table 7 – Micronutrient, creatinine and homocysteine levels

| Unadjusted means ± SD | | | **Baseline** | |  | **Day 1** | | | **Day 28** | | |
| --- | --- | --- | --- | --- | --- | --- | --- | --- | --- | --- | --- |
|  | | N | Mean | SD | Mean | | SD | Mean | | SD |  |
| Ferritin | PLA | 17 | 81.65 | 59.28 | 90.82 | | 58.90 | 76.82 | | 57.63 |  |
|  | MVM | 18 | 69.22 | 43.07 | 75.56 | | 48.13 | 74.72 | | 42.05 |  |
| Vitamin B12 | PLA | 16 | 335.44 | 130.14 | 352.33 | | 136.83 | 359.06 | | 126.64 |  |
|  | MVM | 18 | 349.78 | 123.99 | 366.72 | | 133.48 | 396.67 | | 115.34 |  |
| Creatinine | PLA | 17 | 87.06 | 17.25 | 85.71 | | 19.30 | 92.12 | | 18.17 |  |
|  | MVM | 18 | 88.83 | 10.55 | 89.67 | | 10.37 | 90.94 | | 11.17 |  |
| Homocysteine | PLA | 16 | 10.30 | 9.12 | 10.67 | | 9.60 | 9.19 | | 4.59 |  |
|  | MVM | 18 | 8.65 | 3.21 | 9.27 | | 3.62 | 8.15 | | 3.24 |  |

MVM=Multivitamin-Mineral; PLA=Placebo

Supplementary table 8 – Recovery biomarker levels

| Unadjusted means ± SD | | | **Baseline** | |  |  | **Day 1** | | | **Day 28** | | |
| --- | --- | --- | --- | --- | --- | --- | --- | --- | --- | --- | --- | --- |
|  | | N | Mean | SD | Sample Number | Mean | | SD | Mean | | SD |  |
| GPX | PLA | 15 | 448.25 | 237.52 | 1 |  | |  | 451.17 | | 164.58 |  |
|  |  |  |  |  | 2 | 564.25 | | 277.02 | 476.97 | | 224.68 |  |
|  |  |  |  |  | 3 | 473.16 | | 149.49 | 507.21 | | 297.98 |  |
|  |  |  |  |  | 24 | 516.17 | | 245.71 | 422.06 | | 207.08 |  |
|  |  |  |  |  | 48 | 483.91 | | 330.34 | 423.86 | | 243.76 |  |
|  | MVM | 14 | 523.94 | 252.27 | 1 |  | |  | 623.02 | | 233.57 |  |
|  |  |  |  |  | 2 | 500.12 | | 243.03 | 525.05 | | 125.44 |  |
|  |  |  |  |  | 3 | 509.38 | | 220.44 | 646.92 | | 274.89 |  |
|  |  |  |  |  | 24 | 552.48 | | 240.59 | 551.83 | | 204.72 |  |
|  |  |  |  |  | 48 | 565.98 | | 219.75 | 458.44 | | 237.56 |  |
| Protein Carbonyls | PLA | 15 | 5.82 | 1.58 | 1 |  | |  | 7.88 | | 6.90 |  |
|  |  |  |  |  | 2 | 6.82 | | 3.38 | 8.36 | | 3.13 |  |
|  |  |  |  |  | 3 | 6.49 | | 2.38 | 6.96 | | 2.70 |  |
|  |  |  |  |  | 24 | 22.09 | | 56.00 | 8.66 | | 4.34 |  |
|  |  |  |  |  | 48 | 9.02 | | 10.89 | 6.42 | | 3.99 |  |
|  | MVM | 14 | 7.95 | 3.34 | 1 |  | |  | 8.43 | | 10.82 |  |
|  |  |  |  |  | 2 | 9.33 | | 3.55 | 5.39 | | 7.53 |  |
|  |  |  |  |  | 3 | 7.50 | | 5.63 | 8.81 | | 3.79 |  |
|  |  |  |  |  | 24 | 7.49 | | 4.30 | 5.52 | | 4.83 |  |
|  |  |  |  |  | 48 | 7.26 | | 5.05 | 6.50 | | 4.59 |  |
| 8-Isoprostane | PLA | 15 | 21.04 | 8.87 | 1 |  | |  | 23.12 | | 12.07 |  |
|  |  |  |  |  | 2 | 26.97 | | 10.90 | 23.11 | | 11.62 |  |
|  |  |  |  |  | 3 | 16.52 | | 3.76 | 20.68 | | 9.44 |  |
|  |  |  |  |  | 24 | 16.23 | | 7.31 | 23.19 | | 14.64 |  |
|  |  |  |  |  | 48 | 18.11 | | 9.66 | 17.55 | | 8.36 |  |
|  | MVM | 14 | 33.96 | 17.37 | 1 |  | |  | 27.81 | | 16.30 |  |
|  |  |  |  |  | 2 | 32.63 | | 16.55 | 29.08 | | 12.48 |  |
|  |  |  |  |  | 3 | 28.62 | | 10.70 | 22.02 | | 6.90 |  |
|  |  |  |  |  | 24 | 26.91 | | 14.15 | 23.39 | | 11.28 |  |
|  |  |  |  |  | 48 | 32.73 | | 19.10 | 20.97 | | 11.15 |  |

GPX=Glutathione Peroxidase; IL6=Interleukin-6; MVM=Multivitamin-Mineral; PLA=Placebo
